# Rapid species-level metagenome profiling and containment estimation with sylph

**DOI:** 10.1038/s41587-024-02412-y

**Published:** 2024-10-08

**Authors:** Jim Shaw, Yun William Yu

**Affiliations:** 1 Department of Mathematics, University of Toronto, Toronto, Ontario Canada; 2 Computational Biology Department, Carnegie Mellon University, Pittsburgh, PA USA; 3 Present Address: Department of Data Science, Dana-Farber Cancer Institute, Boston, MA USA; 4 Present Address: Department of Biomedical Informatics, Harvard Medical School, Boston, MA USA

**Keywords:** Classification and taxonomy, Genome informatics, Metagenomics

## Abstract

Profiling metagenomes against databases allows for the detection and quantification of microorganisms, even at low abundances where assembly is not possible. We introduce sylph, a species-level metagenome profiler that estimates genome-to-metagenome containment average nucleotide identity (ANI) through zero-inflated Poisson *k*-mer statistics, enabling ANI-based taxa detection. On the Critical Assessment of Metagenome Interpretation II (CAMI2) Marine dataset, sylph was the most accurate profiling method of seven tested. For multisample profiling, sylph took >10-fold less central processing unit time compared to Kraken2 and used 30-fold less memory. Sylph’s ANI estimates provided an orthogonal signal to abundance, allowing for an ANI-based metagenome-wide association study for Parkinson disease (PD) against 289,232 genomes while confirming known butyrate–PD associations at the strain level. Sylph took <1 min and 16 GB of random-access memory to profile metagenomes against 85,205 prokaryotic and 2,917,516 viral genomes, detecting 30-fold more viral sequences in the human gut compared to RefSeq. Sylph offers precise, efficient profiling with accurate containment ANI estimation even for low-coverage genomes.

## Abstract (web summary)

The microbial composition of metagenomes is identified in seconds by profiling against large databases.

## Main

Shotgun metagenomics, the sequencing of all microbial genomes in a sample, has allowed for unprecedented insight into microbial communities without the need for cultivating those communities in a wet lab^1,2^. Computational analyses often proceed by assembling metagenome-assembled genomes (MAGs) or profiling the reads against a database of reference sequences. While assembly is necessary for novel genome discovery, a fundamental drawback is that assembly may not work for low-abundance organisms. Reference-based profiling methods instead leverage vast collections of microbial genomes^3,4^ to identify microorganisms and their abundances even for low-abundance organisms.

Metagenomes can be complex and large^5–7^. This necessitates accurate profiling methods that scale with high-depth samples and big databases. Algorithmic paradigms that have emerged under these constraints include efficient finding of short, exact matches from reads to a genome database^8–10^ or sensitive read alignment against databases of genes, such as species-specific marker gene databases^11^ or universal marker gene databases^12,13^. Methods that find short, exact matches are known to have high numbers of false positives; therefore, abundance cutoffs and confidence thresholds are often used^14^, especially for Kraken^8^ and its derivatives^10^. Species-specific or universal marker gene methods (hereafter denoted as ‘marker gene methods’ and not to be confused with 16S sequencing) are more precise because they retain less but more relevant information in their database. However, such methods usually use databases that are difficult to build and hard for users to customize.

An alternative algorithmic approach is *k*-mer sketching^15,16^, where *k*-mers are subsampled from sequences using MinHash-derived^17–19^ techniques into ‘bags of *k*-mers’ called a sketch. This compressed representation allows for quick average nucleotide identity (ANI) estimation of any reference genome against the genomes in a metagenome through containment *k*-mer statistics^20^. By contrast, popular profilers such as Kraken or MetaPhlAn do not output nucleotide identity information. Although sketching methods tend to be efficient, previous implementations such as Mash Screen^19^ or sourmash^21^ have ANI estimation biases for low-abundance genomes. This is because of missing *k*-mer content as a result of sequenced reads not fully covering the genome^19^, obfuscating ANI calculation. Thus, arbitrary thresholds are required to identify present genomes, an unsatisfactory solution given the importance of detecting low-abundance microorganisms^22^.

## Previous work

The issue of noisy sequence identity estimation due to low-coverage sequencing has attracted attention in various other contexts. Genome-to-genome divergence estimation using *k*-mers^23,24^ suffers from a similar issue when comparing two low-coverage single-genome sequencing samples. In particular, our model uses similar assumptions to Skmer^23^ but we deal with metagenomes instead of single genomes. Recently, containment ANI-based statistical testing of microorganism presence in metagenomes was investigated using YACHT^25^ but a user-provided coverage threshold parameter is still required. Metagenomics gene presence–absence testing^26^ is a related problem that deals with assembly incompleteness but not necessarily low coverage. Ultimately, none of the aforementioned approaches deal with accurately estimating containment ANI and profiling in a metagenomics setting.

## Our contribution

**Fig. 1:**
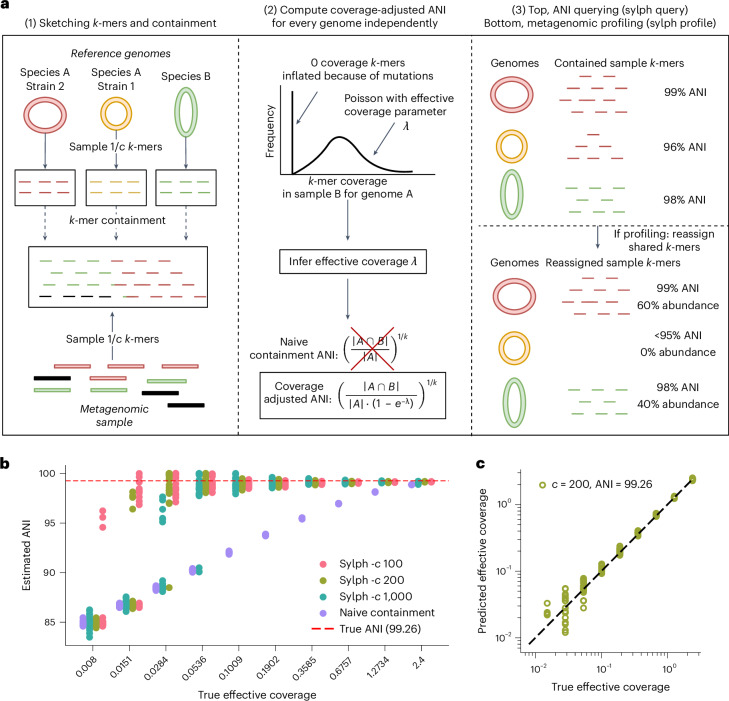
Algorithmic overview of sylph and demonstration for an isolate sequencing run. **a**, (1) Sylph samples $\frac{1}{c}$ of the *k*-mers for metagenomes and reference genomes, resulting in sketches. Black reads are from organisms not present in the database at the species level. Sylph computes the containment of each reference genome’s sketch within the metagenome’s sketch. (2) The coverage distribution of genome A's *k*-mers within metagenome sample B is modeled with a zero-inflated Poisson distribution, where zero inflation is because of divergent *k*-mers between the genome and the metagenome having 0 coverage. Sylph infers the effective coverage *λ* and estimates the coverage-adjusted ANI instead of the commonly used naive ANI, which is ignorant of read-sampling effects. This is repeated for each reference genome against each metagenome sample. (3) Sylph can output the ANI estimates directly (sylph query) or a metagenomic profile (sylph profile), which reassigns shared *k*-mers and reports abundances for genomes with >95% ANI. **b**, Sylph’s ANI estimator over varying *c* (the subsampling rate) for a *K.* *pneumoniae* genome against real, downsampled reads. Effective coverage is shown on the *x* axis in log scale (20 samples per coverage). **c**, Sylph’s predicted effective coverage versus true effective coverage for the *K.* *pneumoniae* samples with *c* = 200 (default).

We present sylph, a *k*-mer sketching metagenomic profiler named for its lightweight and fast characteristics. The key innovation in sylph is a statistical model based on zero-inflated Poisson statistics to debias containment ANI under low coverage (Fig. 1a), solving the low-abundance ANI calculation problem. We show that sylph’s ANI estimation is accurate and apply it to species-level profiling through a principled 95% ANI cutoff^27^.

As a profiler, sylph is unique because it can estimate containment ANI and abundances, thus offering two orthogonal types of information. We display this versatility by finding ANI-based disease-strain associations for Parkinson disease (PD) on large metagenomic cohorts, while also showing that sylph’s species-level taxonomic profiling is both more precise and orders-of-magnitude faster than existing profilers. Sylph’s species-level detection is accurate but, like other profilers, loses sensitivity when organisms only have database representatives at higher taxonomic ranks^28^. Environments with many organisms lacking species-level representative genomes may not be sensitively profiled by sylph but sylph allows for flexible database choice, enabling the usage of massive, growing genome catalogs to potentially improve profiling of undercharacterized microbiomes.

## Results

### Containment ANI estimation by zero-inflated Poisson *k*-mer statistics

Sylph estimates the containment ANI^18^ between a reference genome and a shotgun metagenomic sample by searching the genome against the reads. A mathematical definition of containment ANI can be found in the Methods; intuitively, it measures the similarity of the reference genome to the metagenome and generalizes the standard genome-to-genome ANI.

Sylph first subsamples the *k*-mers (*k* = 31) for each genome in a database of reference genomes or a metagenome sample using FracMinHash^21^, sampling approximately one of *c* *k*-mers (*c* = 200 by default). The collection of subsampled *k*-mers is called a sketch. Sylph then analyzes the containment^20^ of each genome’s sketch in the metagenome’s sketch (Fig. 1a, left).

The key new methodological innovation in sylph is a statistical model to debias the containment computation for low-coverage genomes (Fig. 1a, middle). Under classical stochastic sequencing assumptions^29^, we show that the distribution of a reference genome’s *k*-mer multiplicity in the shotgun metagenome sample follows a zero-inflated Poisson distribution with Poisson parameter *λ* and additional zero inflation. The zero inflation is because of consistent base-level differences between the genome and the metagenome and it is not theoretically affected by random sequencing errors. We call the *λ* parameter in this zero-inflated Poisson the effective coverage; it takes into account sequencing depth, *k*-mer lengths, read lengths and sequencing errors. Sylph attempts to infer *λ* from the distribution and then correct for the missing *k*-mers to obtain a coverage-adjusted ANI estimate. While the containment ANI is known to be slightly biased upward for *k* = 31 compared to standard ANI (Supplementary Fig. 1), this bias subsides for high ANI (>95%); we discuss containment ANI versus standard ANI further in Supplementary Note 1. Therefore, we only work at the species level and refer to containment ANI as just ANI when the context is clear.

After ANI estimation (Fig. 1a, right), sylph can output the ANI results directly using the sylph ‘query’ command. However, this is not a metagenomic profile; a metagenomic profile includes the relative abundances of present taxa but shared *k*-mers between similar genomes obfuscates which genomes are present and their abundance calculation. To resolve this, the sylph ‘profile’ command reassigns shared *k*-mers to the highest ANI genome for each *k*-mer and recomputes coverage-adjusted ANI. Finally, we output the genomes with >95% containment ANI and their abundances after reassignment. Sylph can also estimate the percentage of reads in a sample contained in the database and scale the output abundances.

As a simple test of sylph’s ANI estimator, we took an isolate *Klebsiella pneumoniae* Illumina sequencing run^30^ and downsampled the reads. Under this setup, naive containment ANI (‘naive ANI’ for short; Fig. 1a, middle), which sylph can also output, noticeably underestimates the true containment ANI (99.26% as estimated with all reads) when coverage is <1× (Fig. 1b). However, beginning at even 0.008× effective coverage, sylph can correct ANI to >95% for some samples because of accurate estimation of the effective coverage *λ* (Fig. 1c). Additional synthetic experiments for genomes having 100%, 96% and 90% true containment ANI are shown in Supplementary Figs. 2 and 3. In general, the more similar a genome is to the reference genomes in the database, the better the coverage adjustment becomes.

### Faster and more precise species-level profiling for synthetic datasets

We first constructed a simple undercharacterized synthetic metagenome representing an environment where most of the organisms do not have a species-level representative in the database but have only a genus-level representative. Using the Genome Taxonomy Database (GTDB) R89 (ref. ^31^) database and taxonomy, we created communities with 50 genomes at 95–97.5% ANI and 150 genomes at 85–90% ANI to the nearest ANI genome in the database (Methods). The 50 organisms should be detectable at the species level using the database under the 95% ANI threshold^27^ but the other 150 are only detectable at the genus level. We compared sylph’s profiling against three other methods, Bracken^10^ (with Kraken2)^8^, KMCP (*k*-mer-based metagenomic classification and profiling)^32^ and ganon^9^. This test setup requires a consistent database across the methods, invalidating methods with custom and fixed databases such as MetaPhlAn. In particular, we chose Bracken because of its popularity and KMCP and ganon because they are recent, state-of-the-art methods. All methods were run with default parameters except Bracken, for which we used a 0.01% sequence abundance (as opposed to taxonomic abundance^33^) threshold.

**Fig. 2:**
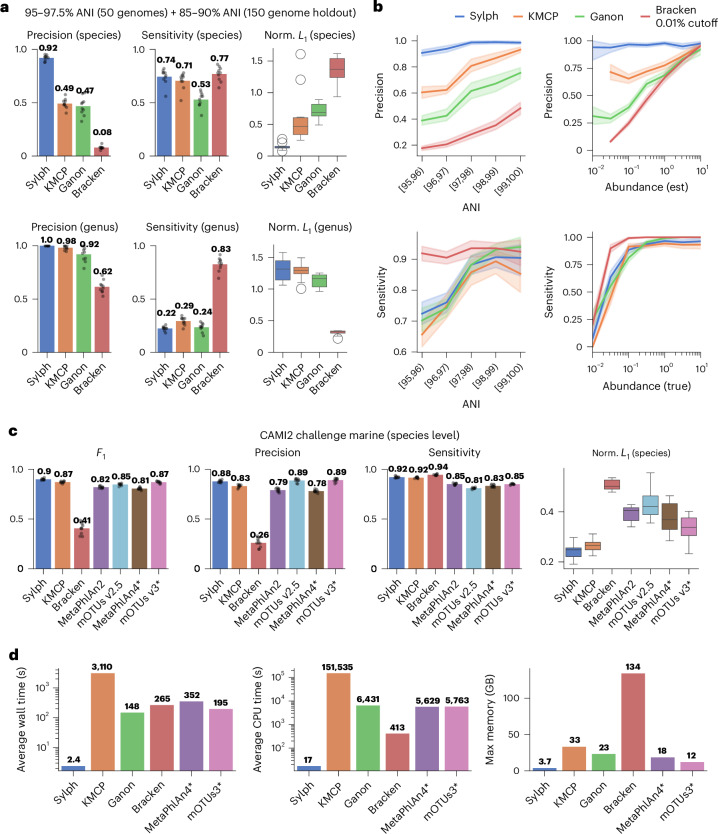
Profiling undercharacterized synthetic metagenomes and the CAMI2 Marine challenge. **a**, Profiling for a synthetic community (ten samples) with 200 genomes: 50 genomes with 95–97.5% ANI to the closest genome in the database and 150 genomes with 85–90% ANI to the closest genome (considered as not present at the species level). All methods used the GTDB-R89 database; a 0.01% abundance cutoff was used for Bracken. Species metrics consider only the 50 genomes with a species representative present. Genus-level results consider all 200 genomes. **b**, Species-level results for five communities with 50 genomes stratified into 1% nearest-neighbor ANI bins (ten samples per bin). The rightmost two plots show the profiling precision and sensitivity over all ANIs (all 50 samples); sequence abundance is binned by 0.5 in log space. The method’s estimated abundance is labeled ‘est’ while the true sequence abundance is labeled ‘true’. **c**, Species-level results on the CAMI2 Marine challenge dataset (ten samples). Methods with asterisks use different databases than the provided CAMI RefSeq database. **d**, Run times and max memory usage for the 200-genome dataset. MetaPhlAn4 and mOTUs3 were run on default databases, whereas other methods used GTDB-R89. Bar plots show mean values and overlaid dots show exact data points. Line plots show mean values and 95% confidence intervals (estimated by bootstrapping). Box plots show the median (middle line), interquartile range (lower and upper box limits) and 1.5 times the interquartile range (whiskers).

Even on this relatively simplistic but undercharacterized synthetic metagenome (Fig. 2a), all methods except for sylph performed poorly for species-level classification, with mean precisions < 50% and *F*_1_ < 60%. On the other hand, sylph had 92% mean precision and 82% *F*_1_. This is likely because of sylph directly using ANI as a cutoff as opposed to a heuristic that only approximates genomic divergence. Sylph also had the lowest normalized *L*_1_ sequence abundance distance at the species level. While sylph outputs species-level abundances and does not attempt higher-level classification, these abundances can be summed to obtain abundances for higher ranks. However, misdetection at lower ranks can skew relative abundances at higher ranks (Supplementary Fig. 4). When benchmarking against all 200 genomes at the genus level, all profilers except for Bracken displayed a high *L*_1_ distance (median > 1) and low sensitivity (mean < 0.32). This includes MetaPhlAn4, despite MetaPhlAn4’s database encompassing GTDB-R89 (taxonomy harmonization described in Methods; Supplementary Fig. 5). However, sylph could accurately estimate the percentage of reads corresponding to the 50 organisms with a species-level database representative (Pearson’s *R* = 0.97; Methods and Supplementary Fig. 6). Ultimately, all profilers except Bracken, which can be unreliable at the species level, were unable to sensitively profile at the genus level (precision–recall curves shown in Supplementary Fig. 7).

To see the effect of genome divergence on profiling, we created five sets of synthetic metagenomes, each containing only genomes within 1% ANI intervals from 95–96% to 99–100% (Fig. 2b) to the closest ANI genome in the database. A striking result is that sylph’s precision was uniquely robust to the ANI between database and sample, maintaining >90% precision over all ANI values. When we grouped all bins together for a uniform [95, 100) dataset (Fig. 2b, right), sylph was the only method that had consistently high precision over all sequence abundances. We also attempted to benchmark against MetaPhlAn4 (Supplementary Fig. 8); while MetaPhlAn4’s precision stayed high across different regimes, its sensitivity and precision were slightly lower than sylph’s. We found that MetaPhlAn4’s species-level genome bin (SGB) definitions made one-to-one taxonomy associations difficult, unfairly lowering its sensitivity and precision on this dataset.

To fairly benchmark against marker gene methods that use fixed taxonomies and databases, we leveraged existing Critical Assessment of Metagenome Interpretation II (CAMI2) challenge^34^ profiler results. These submissions all had access to the same taxonomy and database information. We ran sylph with provided taxonomies and references against a set of CAMI2 submissions for the Marine dataset (Fig. 2c and Methods). We also benchmarked against the Strain Madness dataset and display genus-level results in Supplementary Figs. 9–11. Notably, we ran updated versions of MetaPhlAn (version 4) and mOTUs (version 3) ourselves because existing submissions used outdated versions; however, the new versions use different databases and taxonomy than the official CAMI2 submissions, rendering the comparisons not strictly fair (Supplementary Note 2). For the species-level comparisons, sylph had the highest *F*_1_ score on both the Marine and the Strain Madness datasets. Sylph had the lowest median *L*_1_ error for the Marine dataset and the second lowest for the Strain Madness dataset at the species level, being slightly higher than MetaPhlAn4 (0.16 versus 0.14). Overall, it appeared that sylph was better than or at least comparable to marker gene methods at the species level on the CAMI datasets.

For run time and memory usage, we benchmarked all methods on the 200-genome undercharacterized dataset using 50 threads (Fig. 2d). Sylph was >50 times faster than the next fastest method, ganon, for wall time while taking <4 GB of memory for >25,000 genomes. This was 30 times less than Bracken (134 GB). Sylph was >100 times faster in terms of central processing unit (CPU) time compared to other methods except Bracken (with Kraken2). Kraken2 did not use all cores efficiently; it spent most of the time loading a 134-GB database into memory for each run. On the other hand, sylph is engineered for multisample processing and can sketch and profile many samples at once.

### Sylph’s statistical model generalizes to real short-read and long-read datasets

**Fig. 3:**
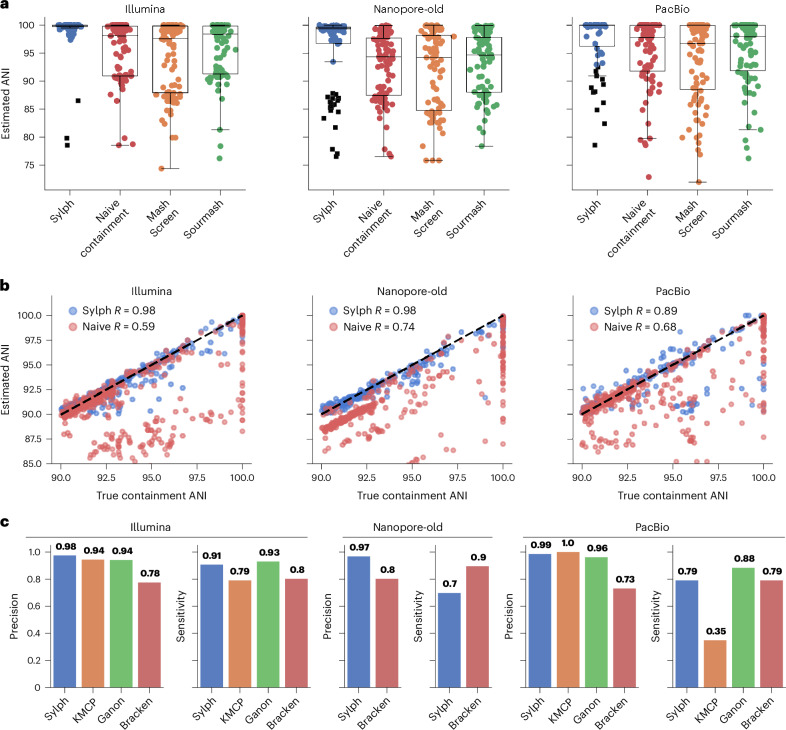
ANI and profiling results for real reads from a mock community downsampled to 1 Gbp. **a**, Each method’s estimated containment ANI for the true reference genomes (87 reference genomes) against the mock community^36^. Black squares indicate genomes whose coverage is too low for sylph’s coverage adjustment; thus, naive ANI is shown instead (these are still included in box plot calculations). Nanopore-old denotes older, less accurate Nanopore reads with 90% sequence identity. Box plots show the median (middle line), interquartile range (lower and upper box limits) and 1.5 times the interquartile range (whiskers). **b**, Sylph’s coverage-adjusted ANI and naive ANI when querying against the entire GTDB-R214 database. The *x* axis is the true containment ANI between a genome in GTDB-R214 and its nearest-neighbor genome in the mock community. The *y* axis is sylph’s estimated ANI. Pearson correlation coefficient values are shown. **c**, Species-level profiling results for the mock community. All methods used the GTDB-R89 database with default parameters except for Bracken, which used a 0.01% abundance cutoff. KMCP and ganon provided no output for the Nanopore reads.

Sylph’s ANI model is based on Poisson coverage statistics but such models may not hold in real samples across different sequencing technologies because of effects such as G+C bias^35^. To evaluate sylph on real reads, we analyzed the 87-genome MOCK2 community from Meslier et al.^36^ sequenced with Illumina, PacBio HiFi (>99% mean identity; Supplementary Note 3) and Oxford Nanopore (older chemistry with 90% mean identity), all downsampled to 1 Gbp (Fig. 3).

Sylph’s ANIs were closer to the true 100% value than Mash Screen and sourmash when querying against a database of true reference genomes (Fig. 3a) and sylph was >6× faster than both (Supplementary Fig. 12). Notably, on the Nanopore dataset, the median ANIs for the naive ANI, Mash Screen and sourmash were all less than 95% because of sequencing errors lowering the *k*-mer containment. However, because sylph can correct for effective coverage, which also takes into account sequencing error, sylph still gave good estimates (>99% median ANI). When using GTDB-R214 as sylph’s database, sylph’s estimated ANIs (from the ‘query’ option, not from the ‘profile’ option) agreed with the containment ANI between the GTDB genome and its nearest-neighbor mock genome (Fig. 3b and Supplementary Fig. 13). This shows that sylph’s coverage-adjusted ANIs can be thought of as a nearest-neighbor ANI query against a reference genome for simpler communities without too much shared *k*-mer content.

Using the same methods and databases for the previous profiling test, we profiled these three sets of reads and found that sylph was the most or second-most performant method on all datasets (Fig. 3c; precision–recall curves shown in Supplementary Fig. 14). The median ANI between the true reference genomes and its corresponding GTDB-R89 nearest-neighbor genome was 100% for this dataset, making the profiling task much easier (Fig. 2b). Notably, sylph and Bracken were the only two methods that worked on the Nanopore dataset by default, whereas the other methods output no results. Thus, sylph works on real reads with real biases; in fact, it works on short, long, low-error and even high-error reads. We attempted to benchmark this community with MetaPhlAn4 (Supplementary Fig. 15) but we found compatibility issues with its SGB taxonomy as in the previous benchmarking attempts. Regardless, MetaPhlAn4 only predicted 72 species in total on the Illumina dataset, whereas sylph and ganon predicted 80 and 81 correct species.

### Comparing against MetaPhlAn4 and mOTUs3 on real, diverse gut metagenomes

Mock and synthetic communities inevitably lack the complexity of real metagenomes. However, previous profiling results (Fig. 2 and Supplementary Figs. 8–11) suggested that sylph’s profiles had similar characteristics to marker gene methods such as MetaPhlAn and mOTUs, including low memory usage and high precision^37–39^ without the need for abundance thresholding^14^. Thus, we investigated the performance of sylph, MetaPhlAn4 and mOTUs3 on real human gut metagenomes without ground truths (database information provided in Methods).

**Fig. 4:**
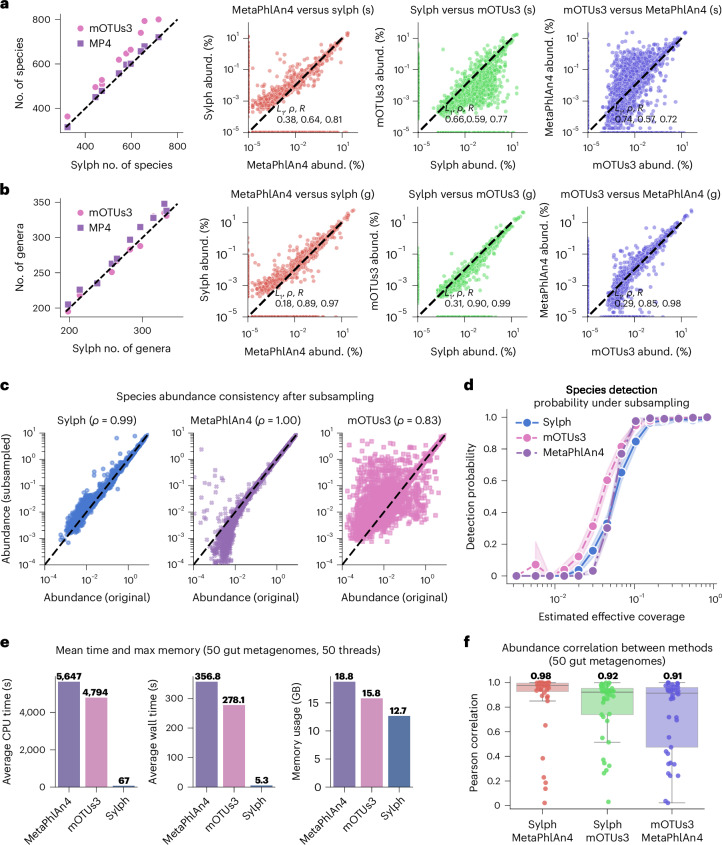
Sylph produces comparable profiles to mOTUs3 and MetaPhlAn4 for diverse human gut samples but is >50× faster. **a**, Species-level profiling results for the ten highest-depth samples from Carter et al.^40^ using mOTUs3, MetaPhlAn4 (MP4) and sylph. Left, the mean number of detected species for each method. Right, taxonomic abundance (abund.) scatter plots for the three two-way comparisons. The mean *L*_1_ abundance distance, Spearman correlation and Pearson correlation are shown. **b**, Genus-level results under the same setup as **a**. **c**, Species-level taxonomic abundance of the Carter et al. samples before and after downsampling by 10×, along with Spearman correlation coefficients. **d**, Probability of species detection after downsampling the Carter et al. samples; effective coverage was determined from the full samples. Mean values and 95% confidence intervals estimated by bootstrapping are shown. **e**, Memory and timing benchmarks for 50 randomly selected human gut metagenomes from GMrepo (version 2)^41^. **f**, Pearson correlation between each method’s species-level abundance estimates on the 50 gut metagenomes. The median is shown above each box plot. The GTDB-R207 database was used in all experiments. Box plots show the median (middle line), interquartile range (lower and upper box limits) and 1.5 times the interquartile range (whiskers).

We first investigated the ten most deeply sequenced metagenomes for an ultrahigh-depth and diverse gut metagenome cohort from Carter et al.^40^ (Fig. 4a–c). Notably, this cohort consists of nonindustrialized gut microbiomes that have high diversity (estimated >700 species on average in sequenced Hadza gut microbiomes). We found that sylph and MetaPhlAn4 detected a similar number of species on average (545 and 554, respectively; Fig. 4a and Supplementary Fig. 16). However, mOTUs3 predicted consistently more species (616 on average). The sylph–MetaPhlAn4 combination also had the lowest species-level and genus-level *L*_1_ distances (Fig. 4b) among all three possible combinations.

Because each of the ten datasets had >50 Gbp sequenced, we further probed the dataset by randomly downsampling the reads by 10×. As a quality-control check, we profiled each method after downsampling and compared it to the original, ‘true’ profiles. Sylph and MetaPhlAn4 produced concordant profiles after downsampling (mean Spearman’s *ρ* = 0.99 and 1.00). However, mOTUs3’s profiles were less consistent after downsampling (Spearman’s *ρ* = 0.83; Fig. 4c), suggesting that sequencing depth affected its abundance estimates. Using the intersection of all three method’s nondownsampled profiles as a true positive dataset, we investigated species detection thresholds (Fig. 4d) and found that mOTUs3 was the most sensitive. Sylph was more sensitive than MetaPhlAn4 at <0.03× effective coverage but MetaPhlAn4 was more sensitive at >0.03× effective coverage.

To investigate a wider variety of sequencing projects, we randomly selected 50 real short-read human gut metagenomes from GMrepo (version 2)^41^ across technologies and time (accessions shown in Supplementary Table 1). On this dataset, sylph was >50× faster than the other two methods in both CPU and wall time (Fig. 4e). As another test, we aligned reads to the database representative genomes of the species detected by sylph over all 50 datasets. We found that sylph gave concordant abundance and coverage estimates to read alignment (Pearson’s *R* = 0.95 and 0.98 for abundance and coverage, respectively; Supplementary Figs. 17 and 18). Overall profiling summary statistics showed similar trends to the Carter et al. dataset; sylph, MetaPhlAn4 and mOTUs3 detected 132, 140 and 152 species and 84, 87 and 91 genera on average over 50 samples, while the sylph–MetaPhlAn4 combination had the highest species-level Pearson correlation (Fig. 4f). In summary, sylph was >50× faster than other state-of-the-art methods while giving similar diversity estimates. For the three possible two-way comparisons of MetaPhlAn4, sylph and mOTUs3, MetaPhlAn4–sylph gave the most similar species-level results.

### Strain-level MWAS for PD

Sylph can estimate containment ANI between metagenomes and databases, giving genome similarity information. Because sylph’s containment ANI estimates work at low coverage, the ANI estimates work even for low-abundance genomes that could not be assembled. The containment ANI of every database genome to the metagenome, obtained with the ‘query’ option, can be viewed as a continuous presence (high ANI) and absence (low ANI) metric of a database’s genomes. Notably, this returns the ANIs at the genome level without collapsing at the species level.

**Fig. 5:**
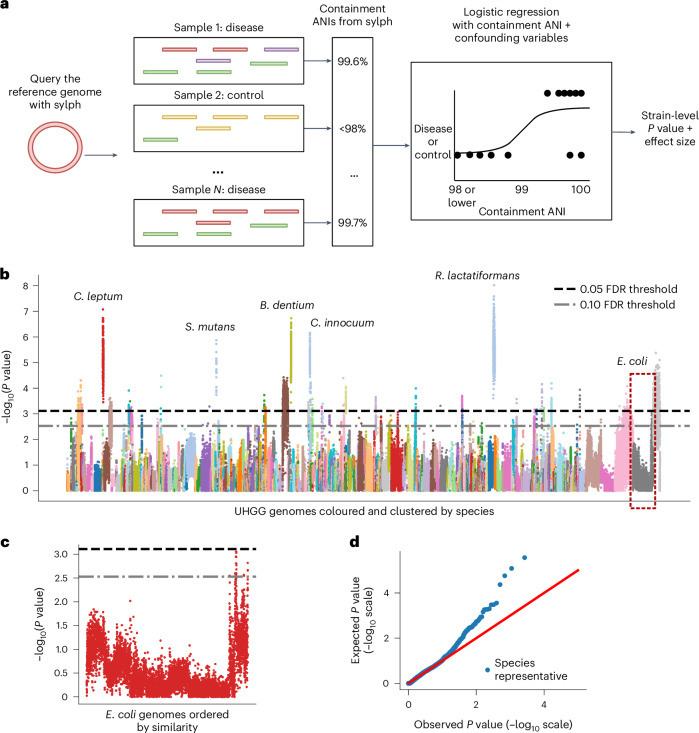
Sylph unveils strain-level ANI–disease associations for PD. **a**, Sylph associates a set of containment ANIs with the query genome against the sample cohort. These containment ANIs are used as covariates for a logistic regression model. The *P* values (calculated by two-sided *z*-tests) for this coefficient and the effect size were used for our associated study. **b**, Manhattan plot for an ANI-based MWAS of 289,232 genomes from the UHGG against 724 gut metagenomes for a PD cohort^42^ colored by species. Five species featuring genomes with low *P* values are labeled and *E.* *coli* genomes are outlined. FDR thresholds at 0.05 and 0.10 were calculated using the Benjamini–Hochberg procedure^44^. **c**, A within-species linear ordering of the genomes was determined by clustering genomes by ANI similarity, unveiling strain-level variation for the *P* values, as shown for *E.* *coli*. **d**, Q–Q plot where only one species representative was picked for each genome.

A study by Wallen et al.^42^ performed a gut metagenome-wide association study (MWAS) for a cohort of 490 PD patients and 234 controls using relative abundance. Instead of relative abundance, we conducted a new MWAS using ANI as a covariate. As opposed to relative abundance, ANI is not compositional (Discussion), allowing for simpler statistical analysis. Furthermore, relative abundance requires collapsing at a taxonomic level, whereas ANI does not. We used a simple logistic regression model for finding associations in two different ways: (1) with a continuous >98% ANI covariate, which we used as the primary method (Fig. 5a), and (2) with a 99% ANI threshold 0–1 variable. We searched for associations among all 289,232 genomes from the Unified Human Gastrointestinal Genome (UHGG) version 2.01 catalog^43^ without dereplicating at the species level. Querying against all 289,232 genomes for 724 samples (5.5 Tbps) took <4 h and 22 GB of random-access memory (RAM) with 40 cores.

In Supplementary Table 2, we display the associations for genomes passing a 0.05 or 0.10 adjusted *P*-value threshold after multiple-testing correction^44^ for methods 1 and 2 described above. In total, 25 and 20 species had a genome passing the 0.05 threshold for methods 1 and 2, respectively, and the intersection had 13 species. The resulting Manhattan plot (only for the >98% ANI method) is shown in Fig. 5b, along with its quantile–quantile (Q–Q) plot in Fig. 5d. To order the genomes in the Manhattan plot, we created a linear ordering on the genomes within a species cluster based on genome similarity (Methods). This linear ordering clustered similar strains together, allowing for visual inspection of the strain-level heterogeneity of *P* values. We show an example for *Escherichia coli* (Fig. 5c); while only 19 of 8,309 *E.* *coli* genomes passed the 0.10 false discovery rate (FDR) threshold, the significant genomes clustered together in a manner analogous to linkage disequilibrium effects in a genome-wide association study (GWAS). We found the passing genomes to be positively associated with PD, in agreement with Wallen et al. We searched the genome with the lowest *P* value against a set of >4,271 *E.* *coli* genomes downloaded from the National Center for Biotechnology Information (NCBI)^27^ and found a 99.99% ANI and 99% aligned fraction match with an *E.* *coli* isolate from a patient with bacteremia (PRJNA203078) and a 99.84% ANI match to a uropathogenic *E.* *coli* strain. Facultative anaerobes such as *E.* *coli* have been noted to be overrepresented in disease states but distinguishing between causality and correlation is difficult^45^. Nevertheless, the interpretability of genome-level results shown here provides an additional avenue for investigating correlations.

Of the 25 genomes passing the 0.05 FDR threshold for the 98% continuous ANI method, five of them were negatively associated with PD. Notably, three of the genomes were *Blautia wexlerae*, *Agathobacter rectalis* (also known as *Eubacterium rectale*) and *Roseburia intestinalis*, which are either producers or are associated with the production of short-chain fatty acids^46–48^ including butyrate. The inverse butyrate–PD correlation was also supported by *Ruthenibacterium lactatiformans* associations, which was enriched in PD and contained the genome with the lowest *P* value. The ratio of *B.* *wexlerae* to *R.* *lactatiformans* abundance was shown to be positively correlated with butyrate production^49^ on a separate PD gut metagenome cohort and *R.* *lactatiformans* presence appears to be inversely correlated with butyrate production in general^50^. *Faecalibacterium prausnitzii*, a prominent butyrate producer^51^, also had a significant genome at 0.10 FDR (but not 0.05 FDR) that was negatively correlated with PD. Ultimately, it appears that using ANI as the main covariate in MWAS leads to a simple but biologically consistent statistical analysis that gives strain-level associations, serving as candidates for further analyses; however, we discuss power limitations in the Discussion.

### Customizing databases for understudied microbiomes, viruses and eukaryotes

A key advantage of sylph over marker gene-profiling methods is that sylph can use arbitrary genome databases as opposed to fixed databases. Sylph does not need taxonomy information, although taxonomy information can be added in postprocessing. This allows for the profiling of prokaryotic MAGs that are not in public databases, as well as eukaryotes and viruses.

**Fig. 6:**
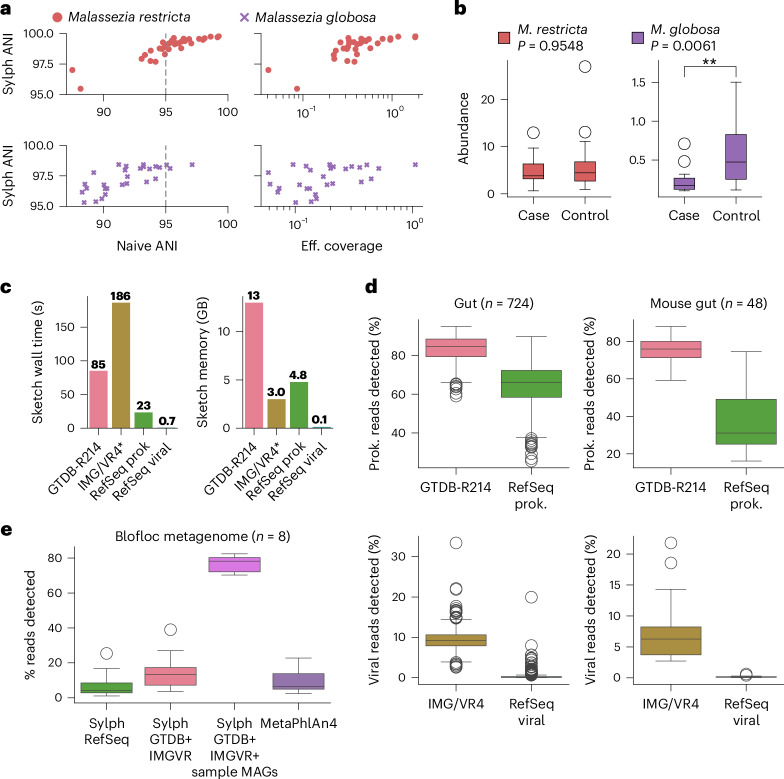
Sylph can improve profiling completeness by incorporating eukaryotes, viruses and arbitrary dereplicated databases. **a**, Sylph’s ANI for two fungal species against skin samples from Chng et al.^52^ as a function of estimated effective (eff.) coverage and naive ANI. **b**, Differential abundance analysis of fungi in case (*n* = 19) and control (*n* = 15) AD skin metagenomes. *M.* *globosa* was significantly differentially abundant (*P* = 0.0061), whereas *M.* *globosa* was not (*P* = 0.9548), similar to conclusions found by Chng et al. *P* values were calculated using two-sided Wilcoxon rank-sum tests. *P < 0.05 and **P < 0.01. **c**, Wall times and max memory usage for sketching databases with 50 threads. **d**, Profiling with RefSeq representative prokaryotic and viral genomes versus GTDB-R214 (ref. ^31^) and IMG/VR4 (ref. ^54^) on human and mouse gut metagenomes. The read detection percentage indicates the percentage of reads that were accounted for by the genomes in the database detected at the species level. **e**, Eight biofloc metagenomes^56^ are not well characterized by existing databases. Sylph can extend existing databases with new genomes and without any additional processing. Adding in MAGs from the eight samples drastically increased the percentage of detected sequences. All box plots show the median (middle line), interquartile range (box boundaries) and 1.5 times the interquartile range (whiskers).

To show that sylph works on eukaryotic genomes, we analyzed skin metagenomes for an atopic dermatitis (AD) disease–control metagenome cohort from Chng et al.^52^ with 19 AD patients and 15 controls. We investigated two prevalent fungal species, *Malassezia restricta* and *Malassezia globosa*. We used sylph to profile all samples against these two genomes and the GTDB-R214 database (accession in Supplementary Table 4). Of the detected *M.* *restricta* genomes, 23% of them were below the 95% naive ANI threshold, whereas 63% of *M.* *globosa* genomes were below the 95% threshold (Fig. 6a), indicating the usefulness of coverage adjustment for low-coverage eukaryotic species. Like Chng et al., who used MetaPhlAn, we found that *M.* *globosa* was differentially abundant between case and control (*P* = 0.0061, Wilcoxon rank-sum test) but not for *M.* *restricta* (Fig. 6b), highlighting the concordance of sylph with other approaches for eukaryote profiling.

To show that sylph can profile viruses precisely, we simulated a viral metagenome from RefSeq and profiled against the Metagenomic Gut Virus (MGV) database^53^ (Methods and Supplementary Fig. 19). Our coverage-adjustment procedure was less sensitive for viruses because of their smaller genome length but the precision for profiling remained high. We then leveraged the IMG/VR4 database^54^, a massive database of viral genomes clustered into 2,917,516 high-confidence operational taxonomic units, and the latest GTDB-R214 database with 85,205 prokaryotic genomes for profiling. Notably, these massive databases only took <300 s and 16 GB of memory to index (Fig. 6c) with 50 threads, implying that sylph is efficient enough to leverage future database growth.

We profiled the Wallen et al. gut samples and a mouse gut metagenome project^55^ (PRJNA549182) with the GTDB and IMG/VR databases, as well as with a database of representative RefSeq prokaryotic and viral genomes (Fig. 6d). Using its ability to estimate the percentage of reads detected at the species level (Methods), sylph detected on average 84.8% of human and 75.1% of mouse gut reads present at the species level in GTDB-R214, whereas 65.2% of human and 37.2% of gut mouse reads were detected at the species level in RefSeq. The effect of virome profiling for RefSeq versus IMG/VR4 was more pronounced, with 9.2% human and 6.9% mouse gut reads being detected as viral on average with IMG/VR4 versus only 0.3% human and 0.2% mouse gut reads with RefSeq.

Many microbiomes are still understudied and are not explained well by databases but sylph allows for easy customization of databases with MAGs to increase detection power. As an example, we took eight recently published biofloc metagenomes^56^ and found that existing databases did not adequately profile the metagenomes at the species level (Fig. 6e). MetaPhlAn4 only detected 9.84% of reads in their database (database version in Supplementary Table 4) on average. By adding in the 444 MAGs assembled from these eight samples to GTDB and IMG/VR, sylph’s average percentage of species-level detected reads increased from 15.5% (only GTDB + IMG/VR) to 76.8% (GTDB + IMG/VR + MAGs). Sylph’s coverage estimates were highly concordant with Burrows–Wheeler Aligner (BWA)^57^ (Supplementary Fig. 20) on these novel MAGs (Pearson’s *R* = 0.995) and, after indexing, sylph’s profiling was >1,000× faster than BWA’s mapping. Thus, sylph can profile and calculate coverages against novel databases with MAGs in a manner concordant with read mapping. In Supplementary Fig. 21, we further show that adding in new catalogs of ocean MAGs and soil MAGs^58,59^ can modestly improve the percentage of detected reads for plant (mean: 25.5–36.1%) and ocean (mean: 17.4–22.8%) metagenomes. Nevertheless, database incompleteness for species-level profiling is still an issue for understudied microbiomes (Discussion).

## Discussion

Sylph is a new method that uses *k*-mer sketching and zero-inflated Poisson statistics for containment ANI computation. When adapted into a metagenome profiler, it is more precise and orders-of-magnitude faster than other methods, especially for multisample profiling. Because of the ANI approach used by sylph for profiling, it has the unique ability to provide ANI estimates for metagenomes, even at low coverages.

The containment ANIs that sylph outputs, providing a continuous measure of presence (high ANI) and absence (low ANI), are in addition to its profiling abilities. We showed that these ANIs give concordant results with previous PD studies based on relative abundance, yet containment ANIs offer genome-centric information at the strain level. Traditionally, relative abundances are used to predict functional implications for a microbiome, as performed in pathway analysis^60^ or differential abundance analysis^61^. However, relative abundances are compositional^62,63^ and are tricky to analyze because of spurious correlations^64^ arising in compositional data. In contrast, containment ANIs are less compositional. An increase in the relative abundance of a species necessarily leads to the lower relative abundance of an unrelated, static species. However, changes in relative proportions should not affect the ANIs because the latter are based on genomic content. We believe that this approach and associated statistical techniques for downstream analysis deserve further exploration. A notable direction to explore is how to handle the loss of power caused by multiple-testing correction in the presence of correlated strains for each species, analogous to the linkage disequilibrium problem in standard GWAS^65^.

While sylph can explain much of the metagenomic reads in well-characterized microbiomes such as the human gut or mouse gut, many environments can still not be classified well at the species level^11^ because of database incompleteness. Our strategy for tackling this problem was to design sylph so that researchers can create customized databases from their novel genomes or MAGs, although this requires the generation of new genomes for researchers working in undercharacterized microbiomes. Despite the continuing efforts to build larger databases, this will be an issue for the foreseeable future given the small fraction of species-level diversity explained by high-quality assembled genomes.

## Methods

### Sketched *k*-mer representation

We represent our sequences and genomes in terms of *k*-mers. We do not use all available *k*-mers in our sequences. Instead, we use a sketch^15^ of all *k*-mers using the FracMinHash method^21^; given a hash function *h* that maps *k*-mers to [0, *M*], we retain a *k*-mer denoted as *x* only if *h*(*x*) < *M*/*c*. The expected fraction of subsampled *k*-mers is exactly $\frac{1}{c}$ under appropriate uniformity and stochasticity assumptions on the hash function; hence, *c* can be thought of as the rate of subsampling. We use minimap2’s^66^ *k*-mer hash function implementation in practice.

Our sketched representation has several benefits. Firstly, we take *c* = 200 by default, effectively speeding up computation and lowering memory costs by a factor of 200. Secondly, two subsampled *k*-mers are probably not near each other in a read or a genome, making them more independent of one another in a statistical sense, as discussed later on.

### Independent substitution model with read sampling

Sylph assumes a generative probabilistic model, where calculating ANI becomes a question of parameter estimation under our model. The model consists of two parts: sequence evolution and read sampling. We consider the independent substitution model without spurious *k*-mers^67^ and uniform random read sampling with error.

Assume we are given a genome *S*. The first part of our random model is an independent substitution model on *S* parameterized by *θ* ∈ [0, 1]; assume every letter in *S* mutates to another letter with probability *θ* independently, resulting in a mutated version denoted *S*′.

#### Definition 1

*Given random substitution parameter θ, let* *τ* = *1* *−* *θ. In our model*, *τ* *is defined as the (fractional) ANI between S and S'*.

Note that we define ANI to be fractional for mathematical convenience.

The second modeling part is a read-sampling step, independent of the first part, where reads are sampled from the mutated genome *S*′ uniformly at random. Each read has some error rate *ϵ*, where errors are also modeled as independent substitutions. Importantly, under the uniformity assumption and that the reads are small enough, we can approximate the depth of coverage as Poisson distributed; if the average number of times a base is covered (its true coverage) is *δ*, the distribution of the number of times a base is covered follows a Poisson distribution with mean *δ*.

We extend this Poisson assumption to *k*-mers. That is, the *k*-mer multiplicity, the number of times a specific *k*-mer is seen, is also Poisson distributed. However, because of the read errors, where every read has its own error rate *ϵ* subject to some distribution *D*_*ϵ*_, the actual coverage of *k*-mers is modified to $\delta \cdot {{\mathbb{E}}}_{\epsilon \sim {D}_{\epsilon }}[{(1-\epsilon )}^{k}]$; a *k*-mer may not be ‘covered’ because of it having an error within the read. Note that random errors are distinct from consistent mutations between genomes. In addition, for a read of length *L*, there are only *L* − *k* + 1 *k*-mers; thus, the coverage decreases by a factor of (*L* − *k* + 1)/*L*. Assuming all reads have length *L*, we provide a second definition.

#### Definition 2

*Assuming read length* *L*, *k*-*mer length* *k*, *coverage δ and read error rate ϵ~D*_*ϵ*_ *under a distribution D*_*ϵ*_*, we define the effective coverage λ as follows*:

$$\lambda =\delta \cdot {{\mathbb{E}}}_{\epsilon \sim {D}_{\epsilon }}[{(1-\epsilon )}^{k}]\cdot (L-k+1)/L.$$

In practice, we observe *k*-mer multiplicities and not the true coverage; hence, the distribution is Poisson with parameter *λ*. That is, sylph only sees the effective coverage under our model and not the true coverage *δ*. We discuss true coverage computation in ‘Computation of effective and true coverage’ (Methods).

### Shotgun metagenome *k*-mer model

Under the above model, we can now precisely state the input and output for sylph. Let *R* be a set of reads from our sample. We model *R* as the output from uniform read sampling for a true underlying set of genomes *G*_1_, *G*_2_, …, *G*_*i*_ at effective coverages *λ*_1_, *λ*_2_, …, *λ*_*i*_, with all final reads mixed together. We model an input genome ${G}_{i}^{{\prime} }$ as mutating from *G*_*i*_ because of random substitutions at ANI *τ*_*i*_. Our goal is to infer and output the true *λ*_*i*_ and *τ*_*i*_ parameters from our *k*-mer data under this model.

#### Definition 3

*Given an input reference genome* ${G}_{i}^{{\prime} }$ *and a fixed hash function resulting in* *N* *FracMinHash k-mers for* ${G}_{i}^{{\prime} }$, *let X*_*1*_, …, *X*_*N*_ *be the multiplicity of* ${G}_{i}^{{\prime} }$’*s k-mers in the reads* *R*.

We make the following assumptions for modeling purposes: (1) the *k*-mers for each genome within the metagenome are unique; (2) if a *k*-mer is mutated in *G*_*i*_*'*, it does not appear in the metagenome; and (3) if a *k*-mer in a read is erroneous, it does not show up in any genome. In practice, assumption 1 is frequently violated (for example, because of mobile elements or repetitive elements). We take these issues into account later in the inference step. Assumptions 2 and 3 are assumed to be reasonable.

Under these assumptions, we derive our model of *X*_*i*_’s distribution. If the *k*-mer for *X*_*i*_ is in *R*, then *X*_*i*_ is Poisson with parameter *λ*_*i*_. However, if the *k*-mer corresponding to *X*_*i*_ is mutated and, thus, differs between *G*_*i*_ and ${G}_{i}^{{\prime} }$, we assume that it will not exist in *R* according to assumption 2; therefore, *X*_*i*_ is 0. The probability of a *k*-mer being not mutated is ${\tau }_{i}^{k}$; hence, it follows that ${X}_{i} \sim {\rm{Pois}}(\lambda ){\rm{Bern}}({\tau }^{k})$, where the Poisson and Bernoulli random variables are independent by assumption. In other words, ${X}_{j} \sim {\rm{ZIP}}({\tau }_{i}^{k},{\lambda }_{i})$, where ZIP is a zero-inflated Poisson distribution. If the sampling locations of the FracMinHash *k*-mers for *X*_*i*_, *X*_*i*+1_, …, *X*_*i*+*N*_ are close together on the genome, these random variables become strongly correlated; nearby *k*-mers will be covered by the same reads frequently. However, we subsample *k*-mers by FracMinHash; therefore, on average, our *k*-mers are *c* bases apart. Hence, we assume that *k*-mers *X*_1_, …, *X*_*N*_ are sufficiently spread out so that the statistical independence of *X*_*i*_ is not a bad assumption in practice.

### Inference for *λ*

We can now rephrase our problem precisely as inferring the parameters *τ*^*k*^ and *λ* from *N* independent and identically distributed (i.i.d.) samples *X*_1_, …, *X*_*N*_ ~ ZIP(*τ*^*k*^, *λ*) for each genome in the database. There exists literature for inference concerning i.i.d. ZIP samples but existing maximum-likelihood estimators and method-of-moments estimators depend on means and variances of *X*_*i*_ (ref. ^68^), which are not robust to long-tailed outliers that are commonly present in metagenomic data (for example, mobile elements and low-complexity *k*-mers). As such, we use a ratio-of-multiplicity estimator for *λ* as used by Skmer^23^, which we define below.

We first define *N*_*a*_ to be the number of *X*_*i*_ with multiplicity *a*. It follows that, for all *a* ≥ 1, ${\mathbb{E}}[{N}_{a}]=N\cdot \Pr ({X}_{i}=a)=N\cdot {\tau }^{k}\cdot ({e}^{-\lambda }{\lambda }^{a})/a!$ Notice that ${\mathbb{E}}[{N}_{a+1}]/{\mathbb{E}}[{N}_{a}]=\lambda /(a+1)$, which does not depend on *τ*. We have samples of *N*_*a*_ available from data; thus, we can use this ratio as our estimator.

#### Definition 4

*The ratio-of-multiplicity* *λ* *estimator was inspired by* *ξ from Sarmashghi* *et al.*^23^. *Our estimator for* *λ* *is as follows*:

1 $$\hat{\lambda }=\frac{{N}_{a+1}}{{N}_{a}}\cdot (a+1).$$

(The estimator used in Skmer^23^ differs from sylph in that their *N*_*a*_ variables are absolute *k*-mer frequencies over all *k*-mers in the sample, whereas our *N*_*a*_ variables are only *k*-mers contained in a reference genome.

In practice, the estimator has reasonable bias except for very small *λ* (Supplementary Fig. 3). We discuss the zero-denominator case in ‘Practical thresholds’ (Methods).

### *λ*-adjusted containment index and ANI

We described how sylph obtains *λ* in the previous section but not the ANI *τ*. The central idea for calculating ANI is simple: check how many *k*-mers from the reference genome are contained in our reads. Let *A* be the set of sketched *k*-mers in our genome and *B* be the set of sketched *k*-mers in our reads. We call $\frac{| A\cap B| }{| A| }$ the containment index^20^. Suppose *A* and *B* are *k*-mers from two complete genomes, without read sampling. In this case, the containment index can be transformed into an estimator for *τ* = ANI by a simple formula, which we call the naive ANI:

$$\frac{| A\cap B| }{| A| }\approx {\tau }^{k}={\rm{AN{I}}}^{k},$$

where the approximate equality can be made precise^18^ under an appropriate random model.

The formula does not take into account coverage and read sampling. It also does not take into account large structural differences between the two genomes, which we discuss further in Supplementary Note 1. To adjust for read sampling, we derive a different ANI formula by the following argument: we can write $| A\cap B| =\mathop{\sum }\nolimits_{i = 1}^{N}I({X}_{i}\ge 1)$, where *I* is the indicator 0–1 random variable. Thus, the expected value is as follows:

$${\mathbb{E}}[| A\cap B| ]=N\cdot \Pr ({\rm{ZIP}}({\tau }^{k},\lambda )\ge 1)=N\cdot [{\tau }^{k}(1-{e}^{-\lambda })].$$

Remembering that we assume a fixed, nonrandom hash function for FracMinHash, whereby ∣*A*∣ = *N*, we get the following:

2 $${\mathbb{E}}\left[\frac{| A\cap B| }{| A| }\right]={\tau }^{k}(1-{e}^{-\lambda }).$$

This immediately gives rise to our final ANI estimator, the *λ*-adjusted containment ANI estimator.

#### Definition 5

*The coverage-adjusted ANI estimate is as follows*:

3 $$\hat{\tau }={\left(\frac{| A\cap B| }{| A| }\cdot \frac{1}{(1-{e}^{-\lambda })}\right)}^{1/k}.$$

We can use this estimator once we know *λ*. In practice, we use the $\hat{\lambda }$ estimate in equation (1) to first estimate *λ*, because this estimate does not depend on *τ*, and then we plug in the estimate $\hat{\lambda }$ into equation (3) to get our final ANI estimate $\hat{\tau }$. Note that the *λ*-adjusted ANI in equation (3) could be >1, in which case we threshold *τ* = 1.

### Practical thresholds

In practice, we apply three filters: (1) we only apply the *λ* adjustment for ANI if the median multiplicity for *k*-mers *X*_1_, …, *X*_*N*_ is ≤3, as the term (1 − *e*^−*λ*^) in equation (3) is small if *λ* ≥ 3; (2) we only proceed with *λ* adjustment if *N*_*a*_ ≥ 3 and *N*_a+1_ ≥ 3, where *a* is the *k*-mer multiplicity that is ≥1 with the most number of *k*-mers (this is usually *a* = 1); these filters are to make sure we only proceed with *λ* adjustment when it is necessary and if enough information is given; and (3) we only output ANIs if the number of FracMinHash *k*-mers for a genome is >50 by default, although this can be changed for smaller genomes and contigs.

### Masking multicopy and dependent *k*-mers

Note that we can remove or mask *k*-mers in *A*, the *k*-mers in our reference genomes, without affecting the inference of our parameters, even if that *k*-mer is in *B*. This masking procedure simply reduces our sample size during inference by discarding some of the sample *X*_*i*_. We can, thus, remove FracMinHash *k*-mers that are not ‘satisfactory’. First, we only take unique FracMinHash *k*-mers in the reference genome, as our *k*-mer Poisson model is predicated on the assumption of unique *k*-mers. Second, we remove FracMinHash *k*-mers in the reference genome if they are less than 30 bp apart by default, as *k*-mers that are too close have strongly correlated coverage, which is not suitable under our assumption of independence. We found that the latter step gives better inferences for small *c*.

### Handling paired-end fragments and duplicated reads

For real short and paired-end reads, we found two major failures of our uniformly distributed read assumption, which in turn impacted our Poisson coverage model: (1) for paired-end reads, if two reads for a pair overlap too much because of a small fragment length^69^, *k*-mers can be double-counted and (2) polymerase chain reaction (PCR) duplicates^70^, such as from Illumina sequencing, can double-count reads and, thus, *k*-mers.

To deal with failure 1, we use a simple heuristic: if a FracMinHash *k*-mer appears twice across a pair of reads, we consider this *k*-mer as counted only once. To deal with failure 2, we use a simple locality-sensitive hashing technique for deduplicating reads that we describe below. In Supplementary Figs. 22–24, we show that deduplication greatly improves detection for low-abundance genomes.

We first explain the deduplication algorithm for paired-end reads. We start by scanning through each read pair and, for each FracMinHash *k*-mer *x* detected, we take the first 32-mer on both the forward read and the reverse read and we mask both 32-mers in a 010101…01 pattern, ending up with two 16-mers that we denote *y*_1_ and *z*_1_. We get another pair of two 16-mers by masking with a 1010…10 pattern instead, which we call *y*_2_ and *z*_2_. We then insert the tuples (*x*, *y*_1_, *z*_1_) and (*x*, *y*_2_, *z*_2_) into a set membership data structure, as discussed below. If one or both of these two tuples are already present, we ignore the FracMinHash *k*-mer and assume that it is because of a PCR duplicate.

The above algorithm is locality sensitive in that, if two PCR duplicates differ by at most one substitution, it is guaranteed to not double-count the *k*-mers coming from a PCR duplicate, as one of the two masking patterns has unchanged bases. In practice, it can often tolerate more. For our set membership data structure, we use a scalable cuckoo filter^71,72^ implementation with false positive rate *P* = 0.0001, which we found to decrease memory consumption while not affecting inference.

For single-end reads, we make a few changes. First, we ignore reads with length > 400 bp, as these are likely to be long reads. Secondly, instead of letting *z*_1_ and *z*_2_ be from the second read in the pair, we take *z*_1_ and *z*_2_ to be the *k*-mers starting from the middle of the read. Finally, we only deduplicate for the FracMinHash *k*-mers with multiplicity < 4, as PCR duplicates are indistinguishable from true reads for high-coverage, single-end read sets.

### Bootstrapping for uncertainty quantification

We found the main source of uncertainty to be in the estimation of *λ* because of read sampling when *λ* is very small, which propagates to uncertainty in *τ*. Other sources of uncertainty, such as because of FracMinHash sampling, have been shown to be small when the ANI is greater than 90% for bacterial genomes^18^.

To quantify the uncertainty in ANI because of *λ* uncertainty, we perform a bootstrap on the resulting distribution of *X*_*i*_ (the *k*-mer multiplicities). Sylph resamples (with replacement) the *k*-mer multiplicities over 100 iterations and performs coverage-adjusted ANI calculations for each iteration. Sylph then takes the 5th and 95th percentile ANI estimates as the 90% confidence interval. Notably, it is possible for a resampling that *N*_*a*_ or *N*_*a*+1_ is 0, in which case we throw out the observation. We only proceed if >50 of the resamples give *N*_*a*_ and *N*_*a*+1_ that are nonzero. Note that our bootstrapping procedure is fundamentally different from the issue encountered in a related study^73^, where bootstrapping causes issues when resampling already-sampled reads; we resample *k*-mers from the reference genome, not *k*-mers from reads.

We show the confidence interval coverage probabilities (‘coverage’ here referring to confidence interval coverage, not read-sampling coverage) in Supplementary Table 3 for a synthetic test. In general, sylph’s confidence intervals are slightly tight but the coverage probabilities are not less than 70% under simulations. Under real samples with more sources of uncertainty, we expect our intervals to be tight but still provide a rough quantification of uncertainty.

### Computation of effective and true coverage

Let *m* be the median *k*-mer multiplicity for a given genome. Effective coverage for a genome is output as either the *λ* estimated from equation (1) if *m* ≤ 3, a robust mean *k*-mer coverage when 4 ≤ *m* ≤ 15 or just *m* otherwise. The robust mean *k*-mer coverage is calculated by taking the mean *k*-mer multiplicity for *k*-mers with multiplicity less than *α*, with *α* defined as follows: *α* is the smallest number such that for Pois(*m*), a Poisson random variable parameterized by *m*, Pr(Pois(*m*) > *α*) < 10^−10^. We found that this robust mean is more accurate than the median for small (effective) coverages because the median is necessarily an integer. However, for large coverages, we found that the robust mean is unnecessary; thus, we use the median instead.

The effective coverage, *λ*, can be much smaller than the true coverage, *δ*, because of read errors and small read lengths (definition 2). Users can toggle sylph to estimate the true coverage instead by using the following method. To estimate *δ*, we need to know *L* and ${{\mathbb{E}}}_{\epsilon \sim {D}_{\epsilon }}[{(1-\epsilon )}^{k}]$, which we denote *E*. This gives the following formula:

$$\delta =\lambda \times \frac{L}{L-k+1}/E.$$

Sylph allows the user to input a fixed read length *L*, otherwise, the mean single-ended read length is used. While read lengths for long reads may be heterogeneous, the effect of read length is negligible for long reads on the effective coverage; hence, this is not an issue in practice. For estimating *E*, if the user inputs a read error rate *ϵ*, sylph simply estimates *E* = (1 − *ϵ*)^*k*^. That is, *D*_*ϵ*_, the distribution of error rates, is a point distribution at *ϵ*. If the user does not input a read error rate, sylph estimates *E* as follows. Defining *n*_*a*_ as the number of *k*-mers in the read sketch with multiplicity *a*, sylph automatically estimates *E* as

$$E=1-\frac{{n}_{1}}{{\sum }_{a\ > \ 1}a\cdot {n}_{a}}.$$

This formula comes from two assumptions: (1) most *k*-mers with multiplicity 1 are erroneous *k*-mers from highly covered genomes and not from lowly covered genomes and (2) erroneous *k*-mers are unique (the same error does not occur more than once). Thus, ∑_*a*>1_*an*_*a*_ ⋅ (1 − *E*) ≈ *n*_1_, giving the above formula. We used sylph’s automatic *E* estimation for all samples unless explicitly stated or if it was from soil or ocean, where *ϵ* = 0.005 (99.5% identity) was assumed. In soil and ocean samples, we found that assumption 1 does not hold and, thus, encourage users to provide an approximate *ϵ* of 0.001–0.010 for short reads instead.

### Profiling by *k*-mer reassignment

After ANI calculation, sylph can output results with the ‘query’ command. However, such results are not a metagenomic profile with accurate relative abundances. Shared *k*-mers within similar genomes in the database may cause multiple related species to have high ANI when only one of the species is actually present.

To reassign shared *k*-mers, we use a winner-take-all heuristic that is also an option in Mash Screen and sourmash. The ‘profile’ command finds putative ANIs in the same manner as ‘query’ but then assigns *k*-mers uniquely to the genome with the highest ANI. For example, if a *k*-mer with a multiplicity of 10 in the sample is present in three database genomes, all ten ‘copies’ of the *k*-mer would be assigned to the single highest containment ANI genome and assumed to not be present in the other two. The ANI computation is then performed again but only considering these reassigned *k*-mers, giving our final ANIs. Sylph then outputs genomes for which ANI is >95% by default.

### Outputting abundances, percentage of reads detected and taxonomic profiles

Let *GL*_1_, …, *GL*_*q*_ be the genome lengths of the *q* genomes passing the threshold. Let *λ*_*i*_ and *δ*_*i*_ denote the effective and true coverage of the *i*th detected genome. Sylph outputs the taxonomic abundance for each genome as ${\lambda }_{i}/\mathop{\sum }\nolimits_{i = 1}^{q}{\lambda }_{i}$. The sequence abundance output is ${\lambda }_{i}\cdot G{L}_{i}/\mathop{\sum }\nolimits_{i = 1}^{q}{\lambda }_{i}\cdot G{L}_{i}$. We estimate the percentage of reads detected at the species level as $\mathop{\sum }\nolimits_{i = 1}^{q}{\delta }_{i}\cdot G{L}_{i}$ divided by the total number of bases in the reads times 100, outputting 100% if this value exceeds 100. Importantly, the percentage of reads detected depends on the true coverage.

We provide a script for turning a metagenomic profile without taxonomy information into a taxonomic profile for multiple prebuilt databases. This script can be easily customized by adding taxonomic accessions to arbitrary genomes. This is achieved by translating each genome in the output to a taxonomic accession string and aggregating sylph’s output abundances at each taxonomic rank and is independent of the core algorithm.

### Synthetic metagenome construction and benchmarking

To construct the undercharacterized synthetic metagenome (Fig. 2a), we first computed nearest-neighbor ANIs from the newer GTDB-R214 database to GTDB-R89. All genome-to-genome ANI calculations were performed with skani^74^. We then arbitrarily selected 50 genomes with 95–97.5% ANI and 150 genomes with 85–90% ANI (Fig. 2a) as nearest-neighbor ANI. Notably, ANI and the aligned fraction between two genomes are positively correlated^75^ (Supplementary Fig. 25), implying that the low-ANI genomes in the synthetic community will likely have large-scale variation not found in our references. We then sampled 3 Gbp of 2× 150-bp reads for this dataset using wgsim^76^ with abundance following a log-normal distribution with mean and s.d. of the underlying normal distribution equal to 2 and 1, respectively, as applied in other benchmarks^38^. Ten samples were created for this dataset. Exact software commands for profiling are shown in Supplementary Note 4. For the five synthetic metagenomes binned by 1% ANIs (Fig. 2b), for each 1% ANI bin, we generated a metagenome with 50 genomes each and 750 Mbp of reads using the same distributions as for the undercharacterized metagenome case.

MetaPhlAn4 could not be fairly profiled against the undercharacterized case because it contains species-level genomes in the genus-level holdout set. We attempted to profile MetaPhlAn4 on the 95–100% binned ANI dataset; its database should contain all GTDB-R89 species as its taxonomy can be mapped to GTDB-R207, a newer database^11^. Therefore, we first mapped taxa from MetaPhlAn4’s default NCBI taxonomy to the GTDB-R207 database as provided by a script included in MetaPhlAn4. Then, we projected the representative genome for the GTDB-R207 taxa to the nearest-neighbor ANI genome’s species name in the GTDB-R89. An issue we found was that MetaPhlAn4 SGBs could collapse species together in the GTDB-R89 taxonomy, thus picking the wrong representative because of the inherent incompatibility with the SGB definition and GTDB. This lowered the precision and sensitivity (Supplementary Figs. 8 and 15).

### CAMI2 benchmarking details

The Strain Madness and Marine challenge datasets from the CAMI2 challenge were used^37^. The Marine dataset consisted of 777 genomes, as well as 200 circular elements. The Strain Madness dataset consisted of 408 genomes with high strain diversity, including over 180 *Streptococcus pneumoniae* strains in a single sample.

To obtain CAMI2 results, we took the profiles for MetaPhlAn (version 2.9), mOTUs (version 2.5) and KMCP as compiled in the KMCP study^32^ and the official CAMI2 repository. We reran all profiles using the latest version of OPAL^34^ (Supplementary Table 4) because we found slightly different values depending on the version of OPAL. For sylph, we took all genomes in the provided RefSeq CAMI2 snapshot (January 8, 2019) with a taxid present and used these genomes as sylph’s database (with default settings). TaxonKit^77^ was used for converting sylph’s output into a CAMI-compatible profile.

We also ran MetaPhlAn4 and mOTUs3 ourselves because no official profiles are available. However, these profilers use more comprehensive databases than the official submissions; thus, direct comparison is not completely fair. We discuss CAMI2 taxonomy issues in Supplementary Note 2.

In the Marine dataset, we found that, for the detected species, many had closely related genomes in the provided database; sylph’s median ANI was 99.8% over all samples for detected genomes in the Marine dataset. There were also species in the true profiles that were not present in the given reference genomes, slightly obfuscating the profiling metrics. For example, *Psychrobacter* sp. JCM 18900 is present in the taxonomy and in the gold-standard profile for multiple samples in the Marine dataset but it is not present in the RefSeq databases and is not found by any method. This causes other *Psychrobacter* species to be profiled and classified as incorrect, lowering the sensitivity and the precision.

### Real metagenome benchmarking with mOTUs3, MetaPhlAn4 and sylph

To fairly compare mOTUs3, sylph and MetaPhlAn4, we used the GTDB-R207 database, which all three methods were compatible with. We directly profiled sylph with the GTDB-R207 database. We used default databases for the other methods (versions in Supplementary Table 4) and mapped their profiles to GTDB-R207 as follows. MetaPhlAn4’s taxonomic profile was mapped to the GTDB-R207 database using the default provided scripts as before. mOTUs3’s output was mapped to GTDB-R207 using the mappings provided at https://zenodo.org/records/10275750. mOTUs3’s corresponding GTDB taxa may have a blank species name; we removed these taxa from species-level comparisons in this case. Effective coverage (Fig. 4d) was estimated by sylph on the full (nondownsampled) metagenomes and divided by 10.

### MWAS procedure details

We ran logistic regression using statsmodels^78^ for each of the 289,232 genomes with sylph’s ANI as a covariate and the disease–control status as the response. In addition, we used all of the same metadata covariates as specified by Table 1 in Wallen et al.^42^ to control for potential confounding (for example, sex and age). Note that we only used one genome per species representative in the Q–Q plot (Fig. 5d) because genomes in the same species give highly dependent *P* values but we ran the MWAS on all 289,232 genomes.

We set a presence–absence threshold of 98% ANI, meaning that, if the ANI for a sample was below 98%, we set it to 98% for the regression. Otherwise, if the ANI was >98%, we kept it as is. This gave a continuous presence–absence covariate so that only high ANIs gave information for the regression, which is desirable when trying to find strain-level associations. We then used the Benjamini–Hochberg procedure^44^ to control for FDR at a significance level of *α* = 0.05.

We created a linear ordering on genomes within a species group, where similar genomes should be placed next to each other along the Manhattan plot, by running an ANI-based average linkage clustering on the genomes. We used skani^74^ for all-to-all ANI calculation and scipy^79^ for clustering, using the resulting dendrogram ordering as the within-species ordering.

### Synthetic viral metagenome profiling

To generate synthetic viral metagenomes for profiling, we downloaded all viral genomes from RefSeq and the MGV database^53^ and computed ANI from all RefSeq viruses to MGV using skani and the ‘-slow’ setting. RefSeq viral genomes with >95% ANI and >80% aligned fraction for both the query and the genome were considered, with the nearest-neighbor MGV genome being designed as the true genome. We randomly selected 50 RefSeq genomes for each sample over ten samples and simulated reads using the same methodology as with the other synthetic metagenomes, except we only sampled 3 Mbp of reads for each sample.

### Benchmarking hardware and software

Benchmarking was performed on an Intel(R) Xeon(R) CPU at 3.10 GHz with 64 cores and 240 GB of RAM as a Google Cloud instance with a solid-state drive. Software versions are shown in Supplementary Table 4.

### Reporting summary

Further information on research design is available in the Nature Portfolio Reporting Summary linked to this article.

## Online content

Any methods, additional references, Nature Portfolio reporting summaries, source data, extended data, supplementary information, acknowledgements, peer review information; details of author contributions and competing interests; and statements of data and code availability are available at 10.1038/s41587-024-02412-y.

## Supplementary information

Supplementary Information Supplementary Notes 1–4, Figs. 1–25 and Tables 3 and 4.

Reporting Summary

Supplementary Tables 1 and 2 Supplementary Tables 1 and 2A–E as separate Excel sheets. Captions for each supplementary table are in the first cell of each sheet.

## Data Availability

GTDB databases^31^ were obtained from https://gtdb.ecogenomic.org/. CAMI2 (ref. ^37^) datasets were taken from https://data.cami-challenge.org/participate, with KMCP^32^ CAMI2 profiling results taken from Zenodo (10.5281/zenodo.7450803)^80^. *K.* *pneumoniae* isolate sequences were previously published^30^ and are available in the Sequence Read Archive under accession number SRR12010075. Meslier et al.^36^ raw data and metadata are available from the European Nucleotide Archive (ENA; PRJEB52977) and additional scripts and references can be found at https://forgemia.inra.fr/metagenopolis/benchmark_mock. Carter et al.^40^ gut metagenomes are available from the ENA (PRJEB49206). Wallen et al.^42^ raw data and metadata are available from NCBI (PRJNA834801). Chng et al. read data are available from NCBI (PRJNA277905) and patient metadata are available in Supplementary Table 4 of the study by Chng et al.^52^ The MGV^53^ and IMG/VR^54^ databases are available at https://portal.nersc.gov/MGV/ and https://genome.jgi.doe.gov/portal/IMG_VR/IMG_VR.home.html, respectively. Mouse gut metagenomes are publicly available from NCBI (PRJNA549182)^55^. Biofloc metagenomes^56^ are publicly available from NCBI (PRJNA967453) and the associated MAGs are available from figshare (10.6084/m9.figshare.23599461)^81^. The 50 real gut metagenomes from GMrepo (version 2)^41^ are available in Supplementary Table 4.

## Code availability

Sylph is available from GitHub (https://github.com/bluenote-1577/sylph)^82^. Data analysis scripts and notebooks can also be found on GitHub (https://github.com/bluenote-1577/sylph-test)^83^.
