# Supplementary material for: Rapid species-level metagenome profiling and containment estimation with sylph
Source: Nat Biotechnol. 2024 Oct 8;43(8):1348–59. doi: 10.1038/s41587-024-02412-y (PMC12339375; doi:10.1038/s41587-024-02412-y)
Supplement: Supplementary file 1 — Supplementary Notes 1–4, Figs. 1–25 and Tables 3 and 4. [file 41587_2024_2412_MOESM1_ESM.pdf]

nature biotechnology

Supplementary information https://doi.org/10.1038/s41587-024-02412-y

Rapid species-level metagenome profiling and containment estimation with sylph

In the format provided by the authors and unedited

Supplementary Note 1: Containment ANI, true ANI, and aligned fraction

Containment ANI versus "true" ANI

In **Methods**, we define ANI mathematically to be a parameter in a simple random mutation model. In particular, it is the probability a base does not mutate. We work with containment ANI, a k-mer based generalization of ANI for comparing genomes against metagenomes. Under our model, containment ANI should equal ANI when the "metagenome" consists of a single genome. However, in practice, there are a plethora of methods for calculating ANI and all produce slightly different estimates [1].

On real genomes, containment ANI can overestimate the true ANI as calculated by standard methods, especially as genomes become more diverged (**Supplementary Fig.** 1). Figure 2 in Ondov et al. [2] also shows that for larger values of k, the length of the k-mers, k-mer based ANIs overestimate the "true" ANI slightly. This would become problematic for genus-level ANI estimates ($< 95\%$ ANI), so sylph only performs species-level profiling with ANI $> 95\%$.

Alignment fraction and containment ANI

Containment ANI is derived from a model that assumes only random substitutions [3, 4], thus containment ANI can not distinguish structural variation (e.g. gene gain/loss) or point mutation. On the other hand, standard ANI methods are based on alignment of orthologous sequences [5] and can distinguish the two types of divergence. In this section, we investigate the effect of alignment fraction, which we will define as the percentage of one genome that is orthologous to the other. Recall Equation 2:

$$\mathbb{E}\left[\frac{|A \cap B|}{|A|}\right] = \tau^k(1 - e^{-\lambda}) = ANI^k(1 - e^{-\lambda}). \tag{4}$$

Here, λ is the effective coverage, which takes into account the coverage, read lengths, and error rates of the reads (see **Definition** 3.2), and A, B are sets of k-mers for a genome and a metagenome. If we know the alignment fraction, we can model its effect by assuming that only a fraction $0 \leq AF \leq 1$ of the k-mers for A have a homologous k-mer in the metagenome B. Alignment fraction is asymmetric, and in this case, this AF represents the fraction of A's genome alignable onto the genomes in metagenome B. The modified formula is now

$$\mathbb{E}\left[\frac{|A \cap B|}{|A|}\right] = AF \cdot \tau^k(1 - e^{-\lambda}) = AF \cdot ANI^k(1 - e^{-\lambda}). \tag{5}$$

After solving for ANI, the modified version of Equation 3 (sylph's ANI estimator formula) is now

$$ANI = \left(\frac{|A \cap B|}{|A|} \cdot \frac{1}{(1 - e^{-\lambda})}\right)^{1/k} \cdot \frac{1}{AF^{1/k}}. \tag{6}$$

This equation states that our original containment ANI estimate (Equation 3) *should* be scaled by a factor of $\frac{1}{AF^{1/k}} > 1$ to estimate the true ANI; in other words, our original formula underestimates the ANI by $AF^{1/k}$. For default k = 31, a 60% ($AF = 0.6$) alignment fraction would imply we are underestimating by a factor of $(0.6)^{1/31} = 0.983$. However, AF is not known a priori and ANI is

what we are trying to solve for, so there are two unknowns in the above equation, and it is not solvable.

Containment ANI bias dominates alignment fraction in practice

On real data, we found that containment ANI *increases* relative to the "true ANI" as alignment fraction decreases (**Supplementary Figure** 1), contradicting the theory. We found that this is because AF is correlated with ANI (**Supplementary Figure** 25), and containment ANI *overestimates* the true ANI as the true ANI becomes smaller (**Supplementary Figure 1**). This overestimation effect dominates the relatively modest decrease in containment ANI due to alignment fraction as derived above.

Supplementary Note 2: CAMI2 profiling with MetaPhlAn4 and mOTUs3

The usage of MetaPhlAn4 and mOTUs3 profiles in our results for the CAMI2 benchmarking were not official submissions for the CAMI2 challenge – to our knowledge, no official submissions for MetaPhlAn4 or mOTUs3 that are concordant with the official CAMI2 databases exist. As such, we reran MetaPhlAn4 and mOTUs3 (in precision mode) using their respective options for outputting results in CAMI format.

Both MetaPhlAn4 and mOTUs3 may contain more genomes in their databases than the standard databases used for the official CAMI2 submissions, so evaluation is not necessarily fair. Furthermore, note that CAMI2 uses the Jan 8, 2019 NCBI taxonomy. While mOTUs3 adheres to this taxonomy [7], MetaPhlAn4 may not (e.g. the Jan21 database uses the Feb 03 2021 NCBI taxonomy: `https://forum.biobakery.org/t/which-ncbi-taxdump-version-used-for-metaphlan4-database/4989`).

Supplementary Note 3: MOCK2 dataset from Meslier et al. [6]

We took a previously published synthetic mock community with 87 known diverse microbial genomes (MOCK2 community from Meslier et al. [6]) sequenced with real PacBio HiFi, Oxford Nanopore R9, and Illumina HiSeq3000 technologies. 1C. Notably, the PacBio HiFi sequencing run appears to have been possibly mislabeled in the study; the authors claim the PacBio run was sequenced from the MOCK1 community but we detected various species not present in MOCK2. For example, *Streptococcus agalactiae* was claimed to be only present in MOCK2, but the PacBio run for MOCK1 had this species detected by sylph at 100% ANI, indicating that MOCK2 was actually sequenced instead.

Supplementary Note 4: Profiling commands and information

We used the GTDB-R89 dereplicated genome database for Fig. 2A,B. We converted the GTDB taxonomy to the taxonomy dump files using `https://github.com/rrwick/Metagenomics-Index-Correction` [8]. We then used each methods method for building their custom database using this taxonomy.

sylph

```
sylph sketch -1 A_1.fq B_2.fq ...  -2 A_2.fq B_2.fq ...  -d sylph_sketches -t 50
sylph profile sylph_sketches/* my_databases.syldb -t 50 > output.tsv
```

Kraken2 + Bracken (0.01% sequence abundance cutoff used)

```
kraken2 --paired --db database --threads 50 r1.fq r2.fq
--report kraken_out/sample.kreport;
bracken -d database -i kraken_out/sample.kreport -o output -r 150
```

KMCP

```
kmcp search -d db_folder/db.kmcp -o sample.kmcp.tsv.gz -1 r1.fq -2 r2.fq -j 50
kmcp profile -X db_folder/ -T db_folder/taxid.map -m 3
sample.kmcp.tsv.gz -o output -C output.profile -s sample_name
```

ganon

```
ganon classify -d ganon_db -p r1.fq r2.fq -o output -t 50
```

MetaPhlAn4

```
metaphlan r1.fq,r2.fq --bowtie2out metaphlan_out/sample.bowtie2.bz2
--nproc 50 --input_type fastq -o output
```

mOTUs v3

```
motus profile -f r1.fq -r r2.fq -t 50 -A -o output
```

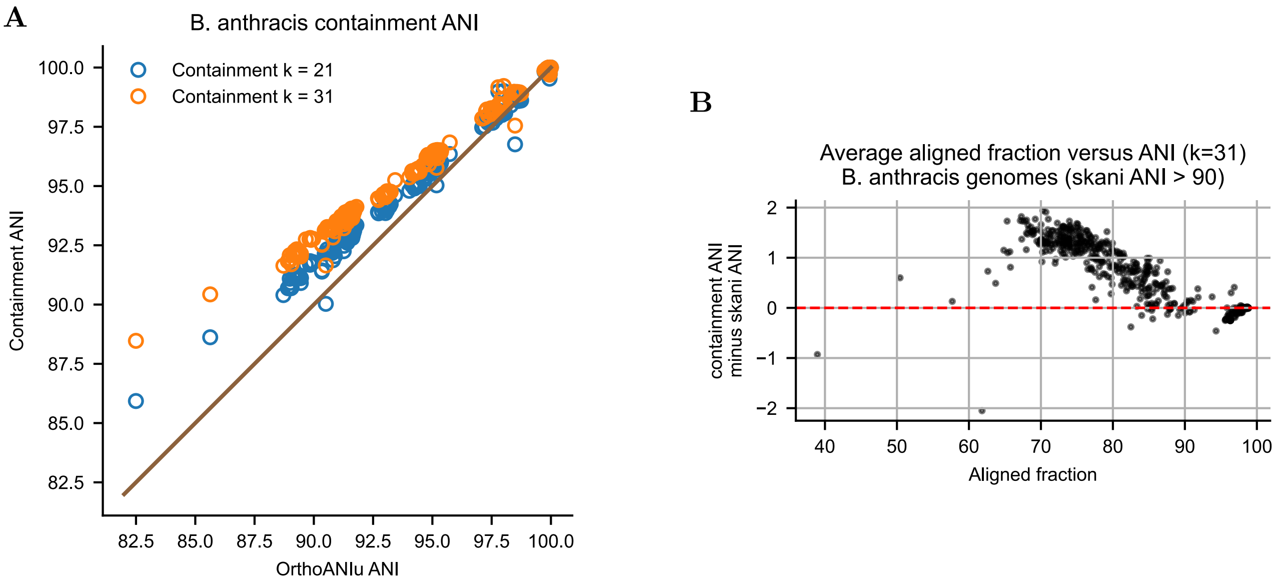

Supplementary Figure 1: **A.** Containment ANI compared to OrthoANI [5] for a *Bacillus anthracis* genome against a database of *B. anthracis* genomes taken dataset D2 from Jain et al. [9]. k=21 or k=31 indicates the k-mer size. **B.** Containment ANI minus skani's ANI [10] as a function of the aligned fraction for the same dataset. Aligned fraction was calculated as the average alignment fraction between the two genomes (alignment fraction is asymmetric).

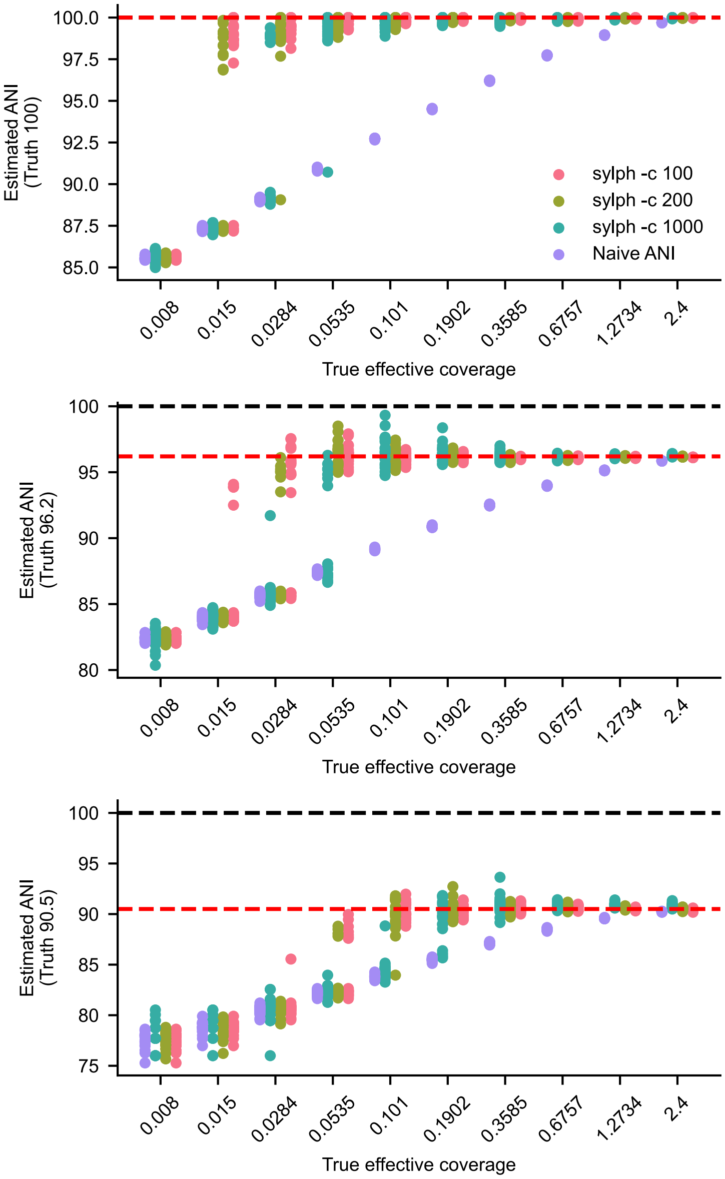

Supplementary Figure 2: Naive containment ANI versus sylph's adjusted ANI estimates when a genome is queried against a set of down-sampled, error-free simulated reads using ART [11]. We simulated 20 sets of reads from *K. pneumoniae* and queried *K. pneumoniae*, *K. africana*, and *K. aerogenes* against the reads from top to bottom subfigures. The true ANI is the true 31-mer containment ANI between *K. pneumoniae* and the respective genome. The coverage adjustment is crucial at lower coverages, and it works better when the true ANI is large and c, the subsampling rate, is smaller. Naive containment ANI calculated with sylph and $c = 1000$. The True ANI is calculated as the 31-mer containment ANI between the two genomes.

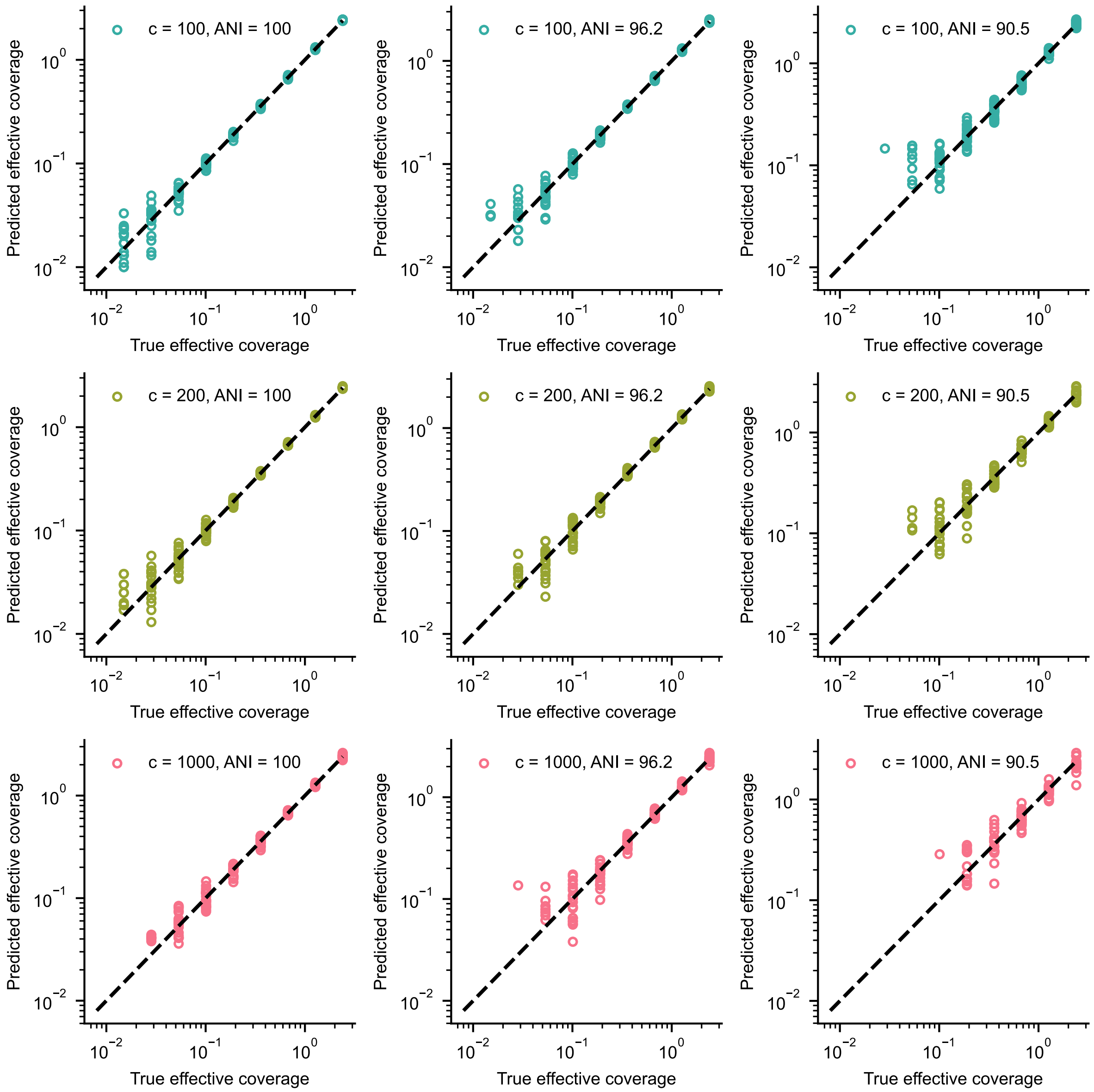

Supplementary Figure 3: sylph's λ (effective coverage) estimator versus the true effective coverage on the same synthetic *Klebsiella* dataset as from Fig. 2. Points were not shown if sylph could not output an estimate for λ.

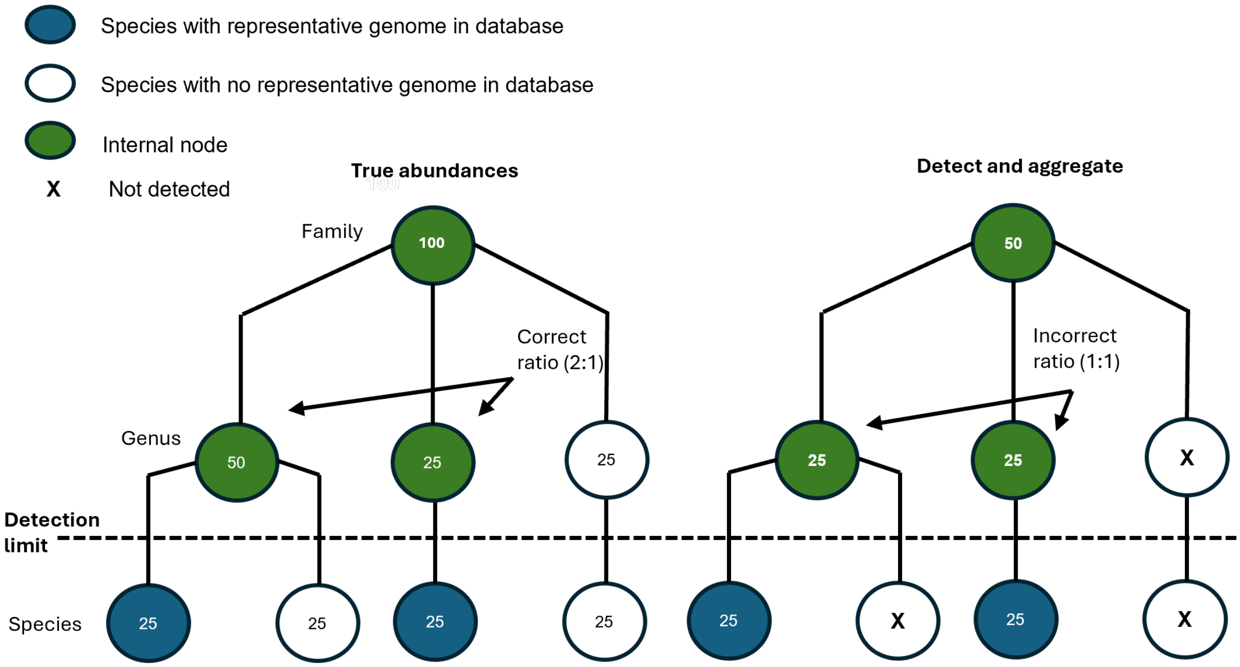

Supplementary Figure 4: Aggregating species-level abundances at higher ranks can lead to inconsistent relative abundances due to misdetection at lower ranks. The true abundances for 4 species are 25% each (left). Some species do not have representative genomes in the database (white). This causes two genomes to be not detected at the species level (right) if a profiler's detection limit is not sensitive enough, leading to incorrect genus-level relative abundances.

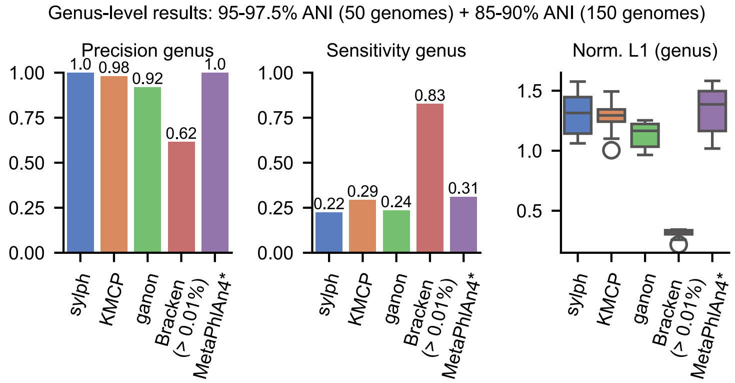

Supplementary Figure 5: The same results as **Fig. 2A** but with MetaPhlAn4 included, using taxonomy harmonization as described in the Methods. Note that MetaPhlAn4 is advantaged in this setup because the Oct22 CHOCOPhlAn database that was used is concordant with GTDB-R207, and thus encompasses GTDB-R89, the database that all other methods are using. Bars show mean values over 10 samples. Box plots show the median (middle line), interquartile range (box boundaries), and 1.5 times the interquartile range (whiskers).

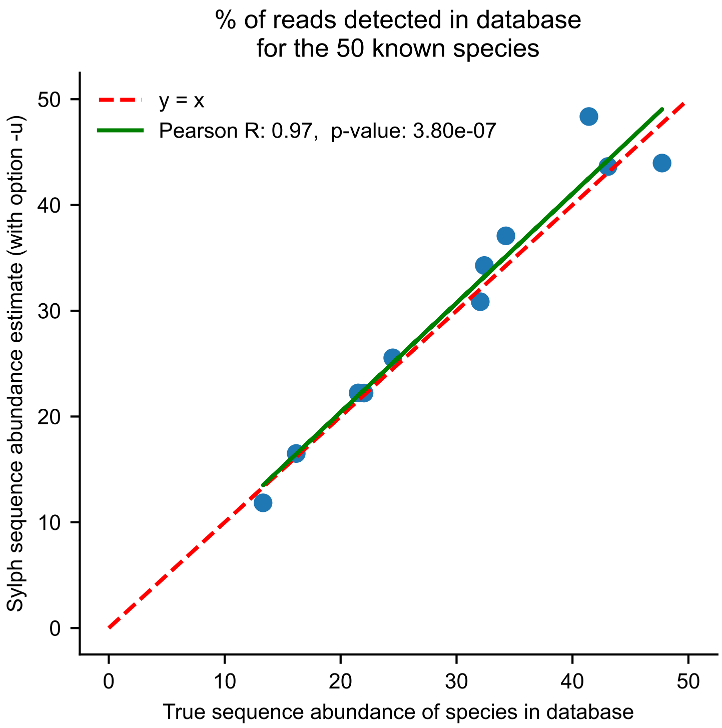

Supplementary Figure 6: Under the same experiment as **Fig. 2A**: sylph's estimates of the percentage of reads corresponding to the 50 genomes (the sequence abundance) with a species-level representative in the database (using the `--estimate-unknown` or `-u` option) versus the true percentage of reads for the 50 genomes with a species-level representative.

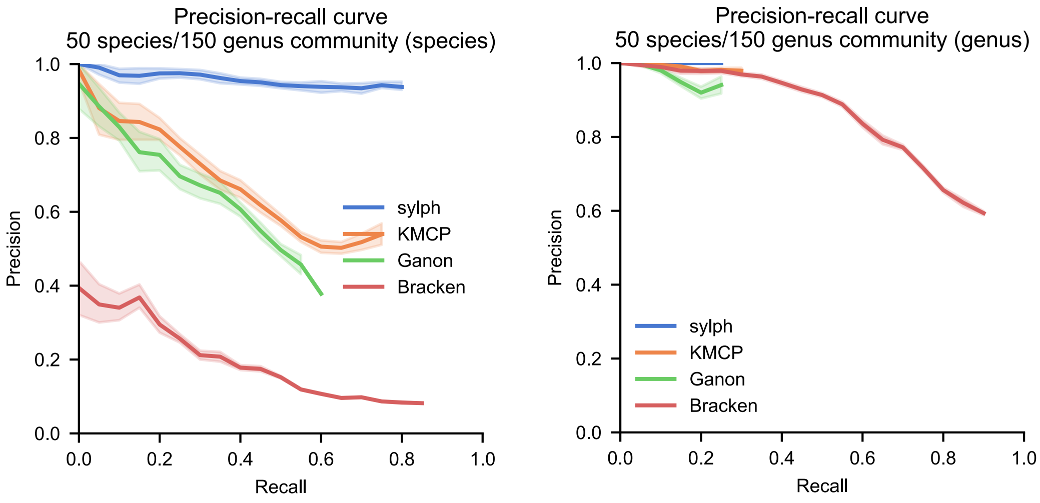

Supplementary Figure 7: Precision-recall curves for the 150-genus 50-species community and methods used for **Fig. 2A** by thresholding at varying levels of abundance. The bar plots shown in Fig. 2A correspond to the rightmost tips of the precision-recall curve. Mean values and 95% confidence intervals estimated by bootstrapping are shown.

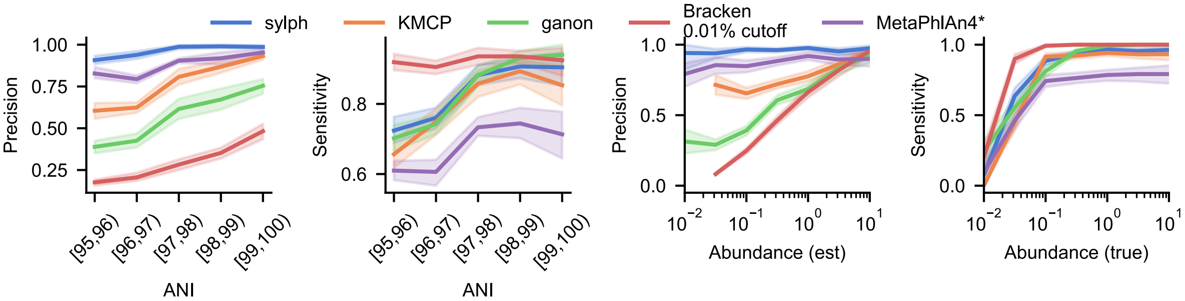

Supplementary Figure 8: MetaPhlAn4 profiling on the combined synthetic metagenomes with 95-100% ANI to the GTDB-R89 database (**Fig. 2B**) with taxonomy harmonization as described in Methods. Mean values and 95% confidence intervals estimated by bootstrapping are shown.

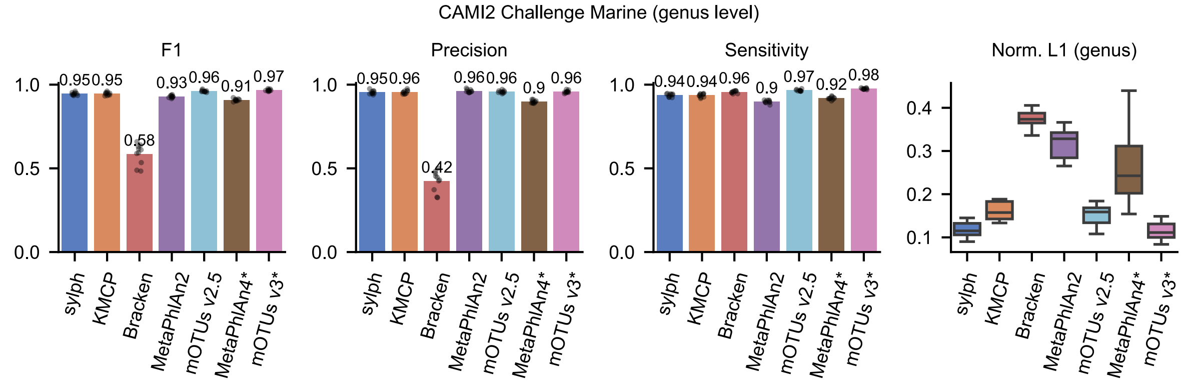

Supplementary Figure 9: Profiling results for the CAMI2 Challenge Marine dataset (10 samples) at the genus level instead of at the species level, including methods that do not use CAMI2's official RefSeq database or taxonomy snapshot (marked with an asterisk). Box plots show the median (middle line), interquartile range (box boundaries), and 1.5 times the interquartile range (whiskers). Bar plots show means with exact values overlayed as dots.

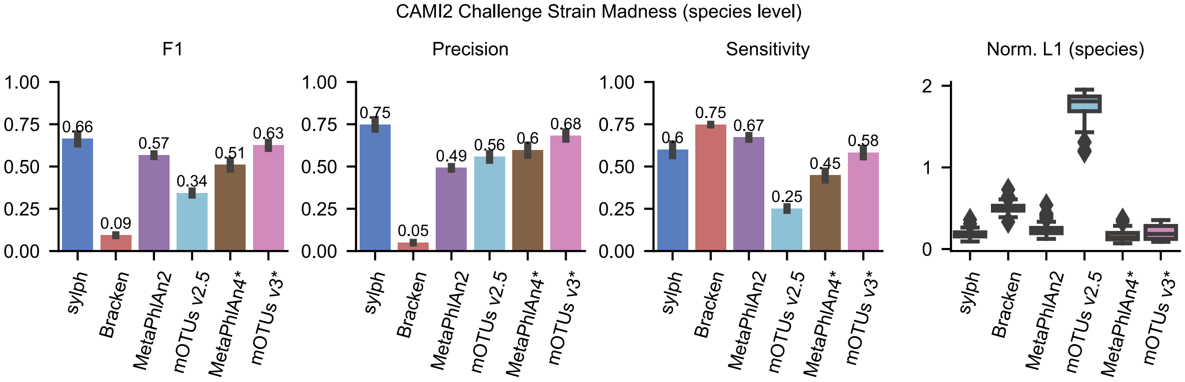

Supplementary Figure 10: Profiling results for the CAMI2 Challenge Strain Madness dataset at the species level, including methods that do not use CAMI2's official RefSeq database or taxonomy snapshot (marked with an asterisk). The first 20 samples from the Strain Madness challenge were used. Box plots show the median (middle line), interquartile range (box boundaries), and 1.5 times the interquartile range (whiskers). Bar plots show mean values with 95% confidence intervals found by bootstrapping.

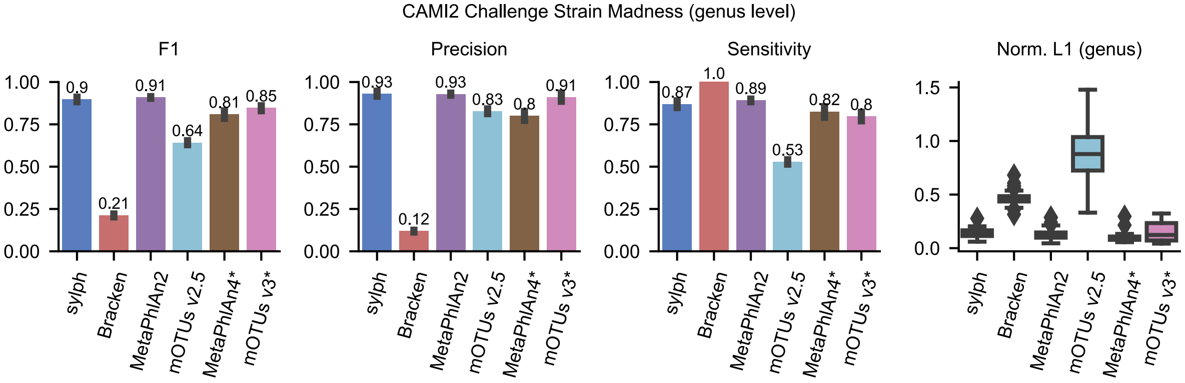

Supplementary Figure 11: Profiling results for the CAMI2 Strain Madness dataset at the genus level instead of at the species level, including methods that do not use CAMI2's official RefSeq database or taxonomy snapshot (marked with an asterisk). The first 20 samples from the Strain Madness challenge were used. Box plots show the median (middle line), interquartile range (box boundaries), and 1.5 times the interquartile range (whiskers). Bar plots show mean values with 95% confidence intervals found by bootstrapping.

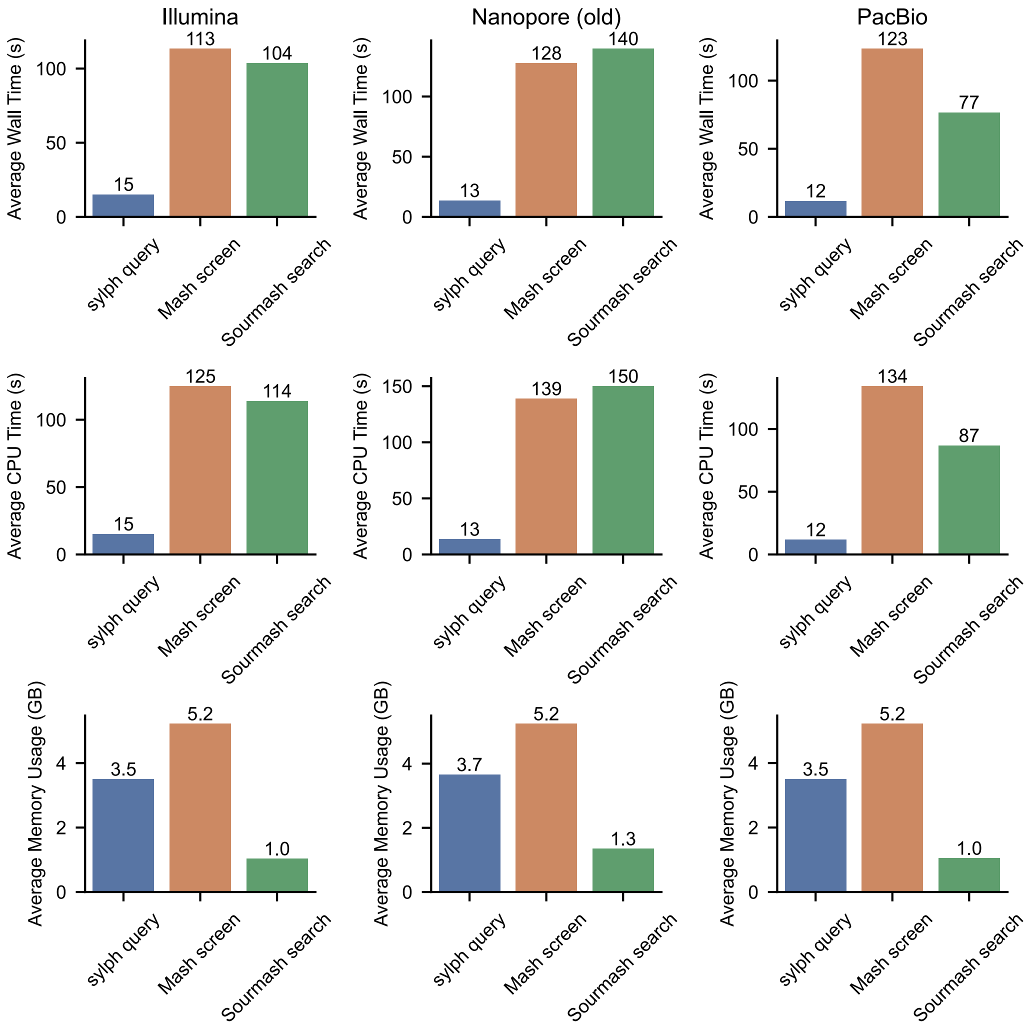

Supplementary Figure 12: Wall times, CPU times, and maximum memory usage of sylph, sourmash, and mash for the three downsampled Meslier et al. datasets. Sketching time was combined into the respective commands shown on the x-axis.

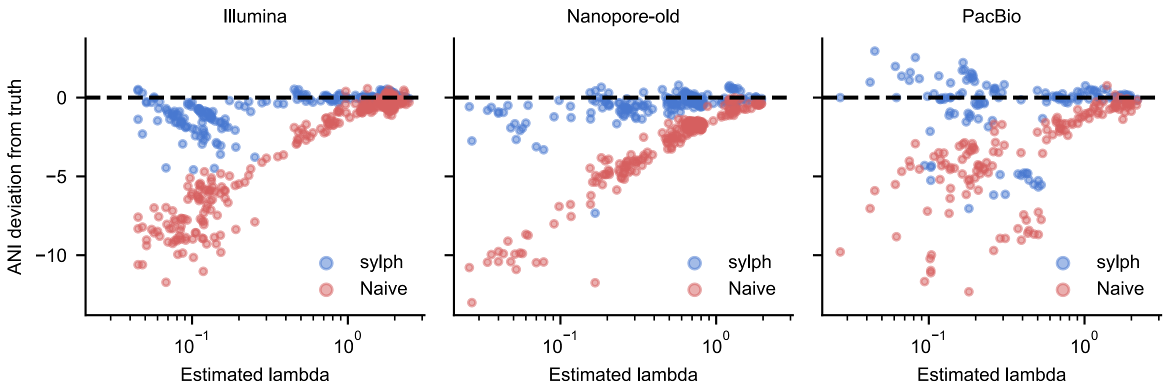

Supplementary Figure 13: Downsampled Meslier et al. [6] dataset queried against the GTDB database. Sylph's ANIs are plotted as deviations from the true nearest neighbour containment ANI as a function of the estimated effective coverage parameter (λ), as estimated by sylph.

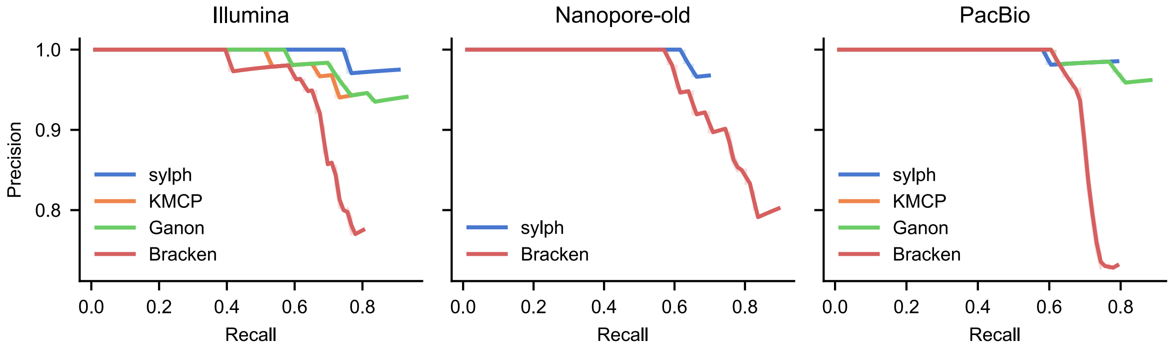

Supplementary Figure 14: Precision-recall curves for profiling of the Meslier et al. dataset (Fig. 4C). Curves were found by thresholding at varying levels of abundance.

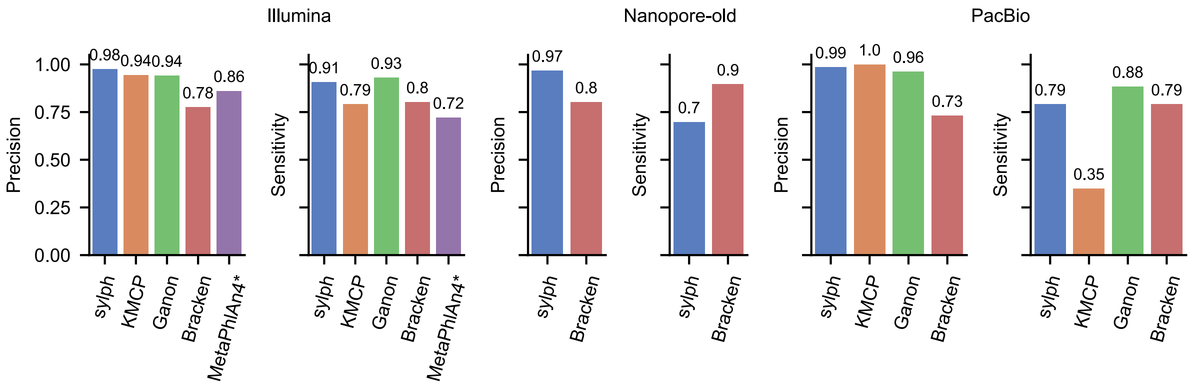

Supplementary Figure 15: Profiling with on the downsampled Meslier et al. database (**Fig. 4C**) but with MetaPhlAn4 included. GTDB-R89 taxonomy harmonization with MetaPhlAn4 is described in Methods.

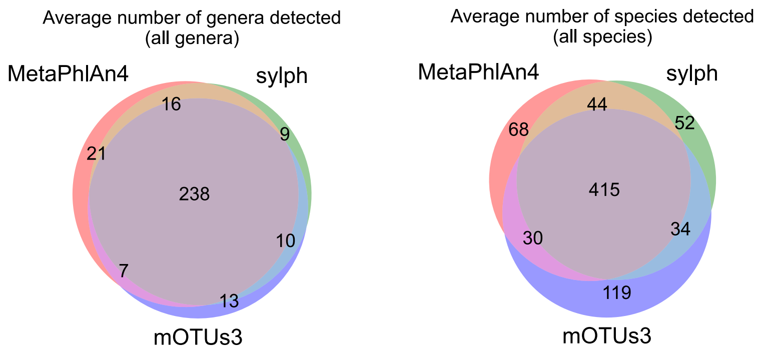

Supplementary Figure 16: Average number of intersecting species and genera from the Carter et al. gut metagenome data set (**Fig. 3A-D**).

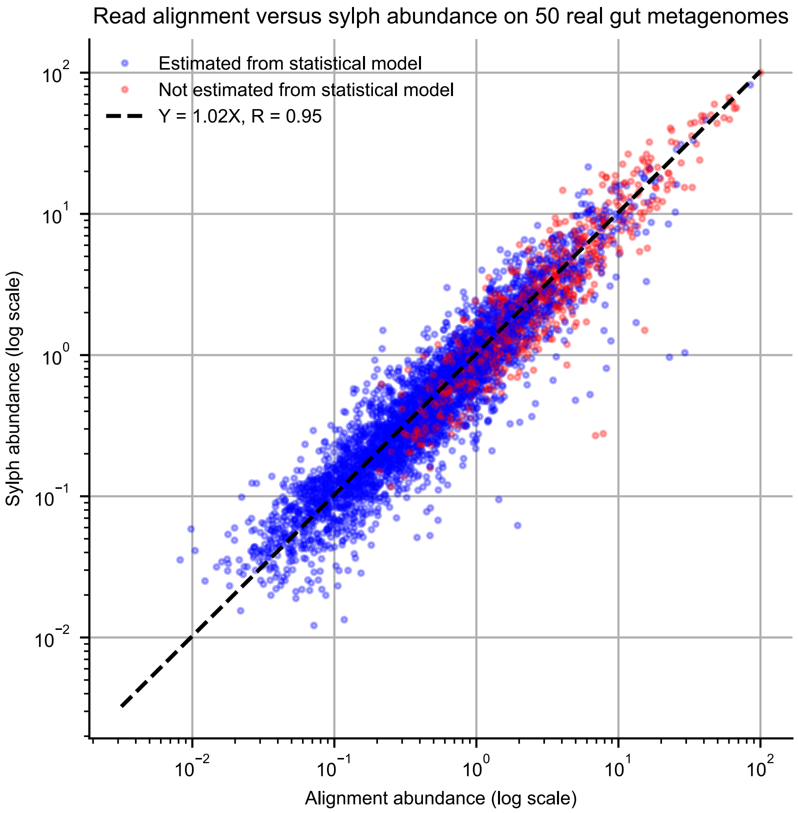

Supplementary Figure 17: Profiling with 50 randomly selected short-read gut metagenomes from GMrepo v2 [12] with sylph and re-aligning the reads to sylph's putatively present genomes to calculate abundances. Coverage was calculated with CoverM [13] and reads were aligned with minimap2 [14]. Blue dots are genomes with coverages estimated by sylph's zero-inflated Poisson model whereas red dots are estimated using medians or means of k-mer multiplicities (see **Methods**).

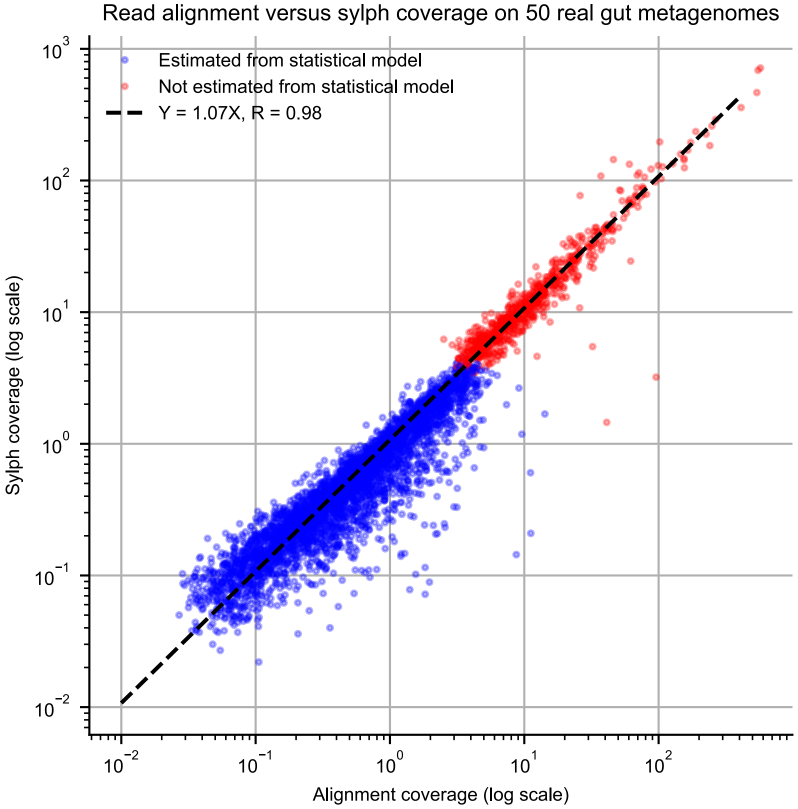

Supplementary Figure 18: The same experiment and dataset as in Supplementary Figure 17 but with coverage instead of abundance shown. Coverage was estimated by arbitrarily assuming a sequence identity of 99.7% across all datasets (needed for estimating true coverage; see **Methods**), corresponding to 0.3% error rate for Illumina reads [15].

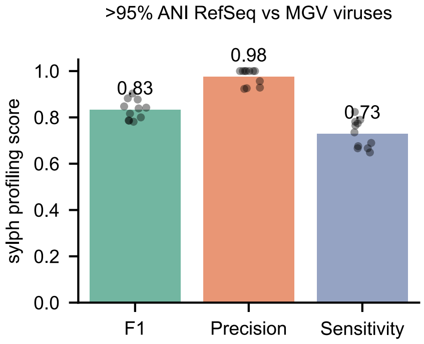

Supplementary Figure 19: Sylph's profiling results for synthetic communities of RefSeq viral genomes against a database of MGV viral genomes [16] with mean values shown and exact values (dots) over 10 samples.

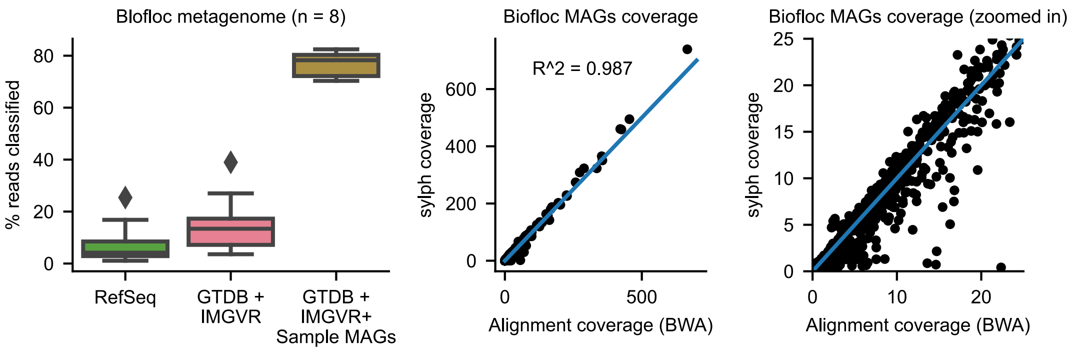

Supplementary Figure 20: Coverage estimates output by sylph (y-axis) for the biofloc MAGs versus coverages obtained from read alignment using BWA (x-axis) with R^2 values for least-squares linear regression is also shown. Box plots show the median (middle line), interquartile range (box boundaries), and 1.5 times the interquartile range (whiskers).

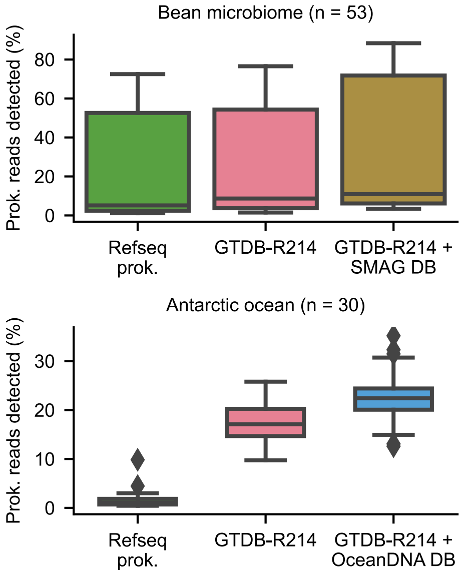

Supplementary Figure 21: Profiling of plant-associated metagenomes [17] (PRJNA904562) and ocean metagenomes [18] (PRJEB61010) against ocean MAGs (OceanDNA) [19] and Soil MAGs (SMAG) [20]. We checked that these metagenomes were made available only after the databases (used by sylph) were released. Box plots show the median (middle line), interquartile range (box boundaries), and 1.5 times the interquartile range (whiskers).

Reference genome (ANI with *K. pneumoniae*)	$c = 100$	$c = 200$	$c = 1000$
K. pneumoniae (100% containment ANI)	78	72	87.
K. africana (96.2% containment ANI)	70	84	91
K. aerogenes (90.5% containment ANI)	83	88	82

Supplementary Table 3: Coverage probabilities of sylph's estimated 90% confidence intervals in Supp Fig. 2, i.e. 100 times the fraction that sylph's 90% confidence interval covered the true ANI over all data points (n = 200). Sylph only outputs a confidence interval when there are enough shared k-mers.

Software/Genome	Description
sylph v0.5.1	Version of slyph.
MetaPhlAn v4.0.6	Version of MetaPhlAn4 with Oct22 CHOCOPhlAn database
Mash v2.3	Version of mash screen.
sourmash v4.8.2	Version of sourmash.
UHGG v2.0.1	Version of UHGG catalogue.
Bracken v2.9	Version of Bracken.
Kraken v2.1.3	Version of Kraken2 used as input to Bracken.
KMCP v0.9.4	Version of KMCP.
ganon v1.9.0	Version of ganon.
opal v1.0.12	Version of opal.
mOTUs v3.1.0	Version of mOTUs.
NZ_CP081897.1	K. pneumoniae genome
NC_015663.1	K. aerogenes genome
NZ_CP084874.1	K. africana genome
NC_040193.1	M. restricta genome
GCA_000181695.1	M. globosa genome
RefSeq prokaryotic/viral database	Representative genomes downloaded on October 2023.
statsmodels v0.14.0	Version of statsmodels used for logistic regression.
scipy v1.10.1	Version of scipy used for average-linkage clustering.
seaborn v0.12.2	Version of seaborn used for plotting/CI calculation.

Supplementary Table 4: Software, genome, and data usage information.

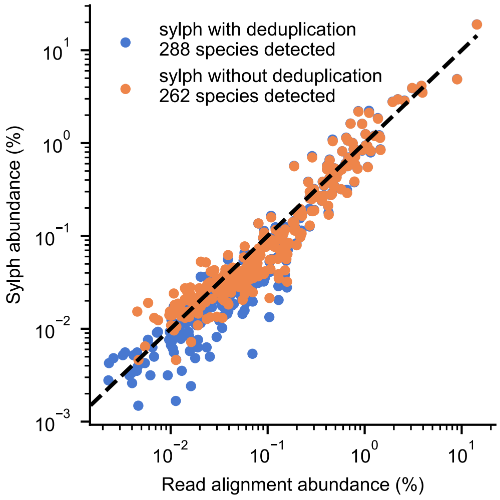

Supplementary Figure 22: Sylph versus read mapping for before and after PCR deduplication for reads (SRR5983472) with 6.33% deduplication rate. Coverage and abundance were calculated using CoverM for the genomes that were present in the deduplicated results.

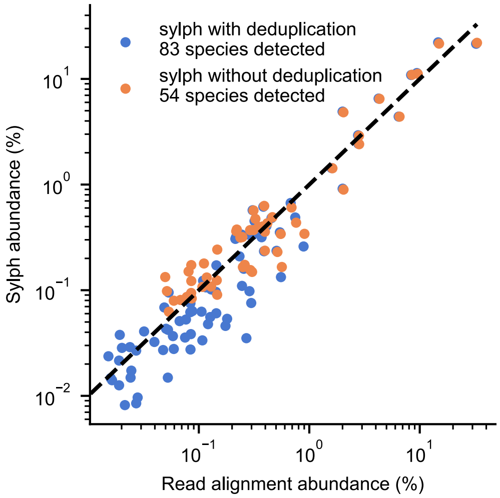

Supplementary Figure 23: Sylph versus read mapping for before and after PCR deduplication for reads (SRR6075232) with 10.94% deduplication rate.

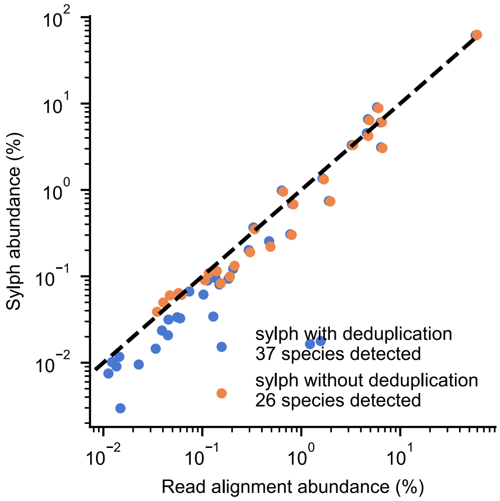

Supplementary Figure 24: Sylph versus read mapping for before and after PCR deduplication for reads (SRR5983313) with 15.91% deduplication rate.

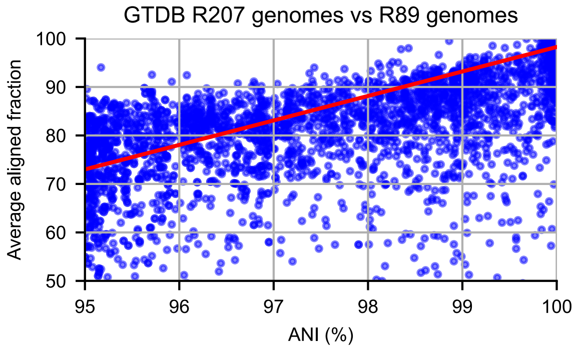

Supplementary Figure 25: The average aligned fraction between two genomes as a function of ANI for the GTDB-R207 database against the GTDB-R84 database as calculated by skani. A robust linear regression, found using the Huber loss function, is shown.

References

[1] Palmer, M., Steenkamp, E. T., Blom, J., Hedlund, B. P. & Venter, S. N. All ANIs are not created equal: Implications for prokaryotic species boundaries and integration of ANIs into polyphasic taxonomy. *International Journal of Systematic and Evolutionary Microbiology* **70**, 2937–2948 (2020).

[2] Ondov, B. D. *et al.* Mash: Fast genome and metagenome distance estimation using MinHash. *Genome Biology* **17**, 132 (2016).

[3] Rahman Hera, M., Pierce-Ward, N. T. & Koslicki, D. Deriving confidence intervals for mutation rates across a wide range of evolutionary distances using FracMinHash. *Genome Research* gr.277651.123 (2023).

[4] Blanca, A., Harris, R. S., Koslicki, D. & Medvedev, P. The Statistics of k-mers from a Sequence Undergoing a Simple Mutation Process Without Spurious Matches. *Journal of Computational Biology* **29**, 155–168 (2022).

[5] Lee, I., Ouk Kim, Y., Park, S.-C. & Chun, J. OrthoANI: An improved algorithm and software for calculating average nucleotide identity. *International Journal of Systematic and Evolutionary Microbiology* **66**, 1100–1103 (2016).

[6] Meslier, V. *et al.* Benchmarking second and third-generation sequencing platforms for microbial metagenomics. *Scientific Data* **9**, 694 (2022).

[7] Ruscheweyh, H.-J. *et al.* Cultivation-independent genomes greatly expand taxonomic-profiling capabilities of mOTUs across various environments. *Microbiome* **10**, 212 (2022).

[8] Méric, G., Wick, R. R., Watts, S. C., Holt, K. E. & Inouye, M. Correcting index databases improves metagenomic studies. *bioRxiv* 712166 (2019).

[9] Jain, C., Rodriguez-R, L. M., Phillippy, A. M., Konstantinidis, K. T. & Aluru, S. High throughput ANI analysis of 90K prokaryotic genomes reveals clear species boundaries. *Nature Communications* **9**, 5114 (2018).

[10] Shaw, J. & Yu, Y. W. Fast and robust metagenomic sequence comparison through sparse chaining with skani. *Nature Methods* 1–5 (2023).

[11] Huang, W., Li, L., Myers, J. R. & Marth, G. T. ART: A next-generation sequencing read simulator. *Bioinformatics (Oxford, England)* **28**, 593–594 (2012).

[12] Dai, D. *et al.* GMrepo v2: A curated human gut microbiome database with special focus on disease markers and cross-dataset comparison. *Nucleic Acids Research* **50**, D777–D784 (2022).

[13] Woodcroft, B. J. CoverM (2023). `https://github.com/wwood/CoverM`

[14] Li, H. Minimap2: Pairwise alignment for nucleotide sequences. *Bioinformatics* **34**, 3094–3100 (2018).

[15] Stoler, N. & Nekrutenko, A. Sequencing error profiles of Illumina sequencing instruments. *NAR Genomics and Bioinformatics* **3**, lqab019 (2021).

[16] Nayfach, S. *et al.* Metagenomic compendium of 189,680 DNA viruses from the human gut microbiome. *Nature Microbiology* **6**, 960–970 (2021).

[17] Mendes, L. W. *et al.* Impact of the fungal pathogen Fusarium oxysporum on the taxonomic and functional diversity of the common bean root microbiome. *Environmental Microbiome* **18**, 68 (2023).

[18] Ilicic, D., Ionescu, D., Woodhouse, J. & Grossart, H.-P. Temperature-Related Short-Term Succession Events of Bacterial Phylotypes in Potter Cove, Antarctica. *Genes* **14**, 1051 (2023).

[19] Nishimura, Y. & Yoshizawa, S. The OceanDNA MAG catalog contains over 50,000 prokaryotic genomes originated from various marine environments. *Scientific Data* **9**, 305 (2022).

[20] Ma, B. *et al.* A genomic catalogue of soil microbiomes boosts mining of biodiversity and genetic resources. *Nature communications* **14**, 7318 (2023).
