# Supplementary material for: Rapid species-level metagenome profiling and containment estimation with sylph
Source: Nat Biotechnol. 2024 Oct 8;43(8):1348–59. doi: 10.1038/s41587-024-02412-y (PMC12339375; doi:10.1038/s41587-024-02412-y)
Supplement: Supplementary file 2 — Reporting Summary [file 41587_2024_2412_MOESM2_ESM.pdf]

nature portfolio

Corresponding author(s): Yun William Yu
Jim Shaw

Last updated by author(s): Aug 16, 2024

Reporting Summary

Nature Portfolio wishes to improve the reproducibility of the work that we publish. This form provides structure for consistency and transparency in reporting. For further information on Nature Portfolio policies, see our Editorial Policies and the Editorial Policy Checklist.

Statistics

For all statistical analyses, confirm that the following items are present in the figure legend, table legend, main text, or Methods section.

n/a	Confirmed	
☐	☒	The exact sample size (*n*) for each experimental group/condition, given as a discrete number and unit of measurement
☐	☒	A statement on whether measurements were taken from distinct samples or whether the same sample was measured repeatedly
☐	☒	The statistical test(s) used AND whether they are one- or two-sided<br>*Only common tests should be described solely by name; describe more complex techniques in the Methods section.*
☐	☒	A description of all covariates tested
☐	☒	A description of any assumptions or corrections, such as tests of normality and adjustment for multiple comparisons
☐	☒	A full description of the statistical parameters including central tendency (e.g. means) or other basic estimates (e.g. regression coefficient) AND variation (e.g. standard deviation) or associated estimates of uncertainty (e.g. confidence intervals)
☐	☒	For null hypothesis testing, the test statistic (e.g. *F*, *t*, *r*) with confidence intervals, effect sizes, degrees of freedom and *P* value noted<br>*Give P values as exact values whenever suitable.*
☒	☐	For Bayesian analysis, information on the choice of priors and Markov chain Monte Carlo settings
☒	☐	For hierarchical and complex designs, identification of the appropriate level for tests and full reporting of outcomes
☐	☒	Estimates of effect sizes (e.g. Cohen's *d*, Pearson's *r*), indicating how they were calculated

Our web collection on statistics for biologists contains articles on many of the points above.

Software and code

Policy information about availability of computer code

Data collection	No software was used for data collection.
Data analysis	sylph v0.5.1 was used throughout this manuscript. Sylph is available at https://github.com/bluenote-1577/sylph and is open-source under the MIT license.MetaPhlAn v4.0.6 was used along with the Oct22 CHOCOPhlAn databaseMash v2.3 was benchmarked against. sourmash v4.8.2 was benchmarked against. Bracken v2.9 was benchmarked against. Kraken v2.1.3 was used prior to running Bracken. KMCP v0.9.4 was benchmarked against. ganon v1.9.0 was benchmarked against. opal v1.0.12 was used to compare CAMI2 profiles to the gold standards for benchmarking. mOTUs v3.1.0 was benchmarked against. Jupyter notebook v7.0.6 was used for interactive plotting and visualization. wgsim v1.20 was used for simulating reads. statsmodels v0.14.0 Version of statsmodels used for logistic regression. scipy v1.10.1 Version of scipy used for average-linkage clustering. seaborn v0.12.2 Version of seaborn used for plotting/CI calculation. bwa v0.7.17 was used for read alignments for coverage calculations. skani v0.2.1 was used for computing genome-to-genome similarity. minimap2 v2.24's hash function was used for developing sylph. OrthoANI (https://www.ezbiocloud.net/tools/orthoaniu) was used for genome-to-genome similarity. Plotting and analysis scripts for all figures are available at https://github.com/bluenote-1577/sylph-test.

For manuscripts utilizing custom algorithms or software that are central to the research but not yet described in published literature, software must be made available to editors and reviewers. We strongly encourage code deposition in a community repository (e.g. GitHub). See the Nature Portfolio guidelines for submitting code & software for further information.

Data

Policy information about availability of data

All manuscripts must include a data availability statement. This statement should provide the following information, where applicable:

- Accession codes, unique identifiers, or web links for publicly available datasets
- A description of any restrictions on data availability
- For clinical datasets or third party data, please ensure that the statement adheres to our policy

GTDB databases [64] were obtained from https://gtdb.ecogenomic.org/. CAMI2 [36] datasets were taken from https://data.cami-challenge.org/participate, with KMCP [31] CAMI2 profiling results taken from https://doi.org/10.5281/zenodo.7450803. K. pneumoniae isolate sequences are previously published [30] and available in the sequencing read archive under the accession SRR12010075. Meslier et al. [35] raw data and metadata are available at PRJEB52977 and additional scripts and references can be found at https://forgemia.inra.fr/metagenopolis/benchmark_mock. Carter et al. [39] gut metagenomes are available at PRJEB49206. Wallen et al. [41] raw data and metadata are available at PRJNA834801. Chng et al. read data is available at PRJNA277905 and patient metadata is available in Supplementary Table 4 of the study by Chng et al [50]. The MGV [51] and IMGVR [65] databases are available at https://portal.nersc.gov/MGV/ and https://genome.jgi.doe.gov/portal/IMG_VR/IMG_VR.home.html. Mouse gut metagenomes are publicly available with accession PRJNA549182 [52]. Biofloc metagenomes [53] are publicly available with accession PRJNA967453, and the associated MAGs are available at https://doi.org/10.6084/m9.figshare.23599461. 50 real gut metagenomes from GMrepo v2 [40] are available in Supplementary Table 4.

Research involving human participants, their data, or biological material

Policy information about studies with human participants or human data. See also policy information about sex, gender (identity/presentation), and sexual orientation and race, ethnicity and racism.

Reporting on sex and gender	N/A
Reporting on race, ethnicity, or other socially relevant groupings	N/A
Population characteristics	N/A
Recruitment	N/A
Ethics oversight	N/A

Note that full information on the approval of the study protocol must also be provided in the manuscript.

Field-specific reporting

Please select the one below that is the best fit for your research. If you are not sure, read the appropriate sections before making your selection.

☒ Life sciences ☐ Behavioural & social sciences ☐ Ecological, evolutionary & environmental sciences

For a reference copy of the document with all sections, see nature.com/documents/nr-reporting-summary-flat.pdf

Life sciences study design

All studies must disclose on these points even when the disclosure is negative.

Sample size	For benchmarking, we evaluated our data using 10 highly divergent samples in Fig. 2A, 50 stratified samples in Fig. 2B, and 10 gold-standard benchmark samples (CAMI2) in 2C. We tested one Illumina, PacBio, and Nanopore dataset in Fig. 3 respectively. We investigated 10 complex real samples in Fig. 4A-D, as well as 50 real samples in Fig. 4E/F.<br><br>Overall, we used over 100 samples over a wide range of designs to test our algorithm, which is enough to demonstrate the effectiveness of an algorithm.<br><br>For Fig. 5 and 6, we analyzed existing datasets (724 Parkinson's metagenomes, 34 skin metagenomes, 48 mouse gut metagenomes, 8 biofloc metagenomes) whose sample sizes were chosen based on previous studies constraints. This was a highly diverse and deep dataset (> 3 terabytes of data) that was analyzed -- enough to show the ability of our algorithm to operate on big data.
Data exclusions	No data was excluded from analysis.
Replication	All algorithms are deterministic and can be downloaded, as well as being open source. All analysis of experiments and reproduction of figures can be found in https://github.com/bluenote-1577/sylph-test.
Randomization	Randomization is not applicable to our synthetic benchmarks of our algorithm since the data is synthetic.<br><br>For real biological data analyzes, all data was previously published and experimental groups were allocated in previous studies. For example,

	the PD cohort was from a previous study by Wallen et al. We do not re-distribute cohorts during our analysis. For MWAS, covariates were controlled for by using the exact same methodology (including a set of chosen covariates as regression variables) as outlined in previously published studies
Blinding	Not applicable. Blinding is not applicable for designing synthetic benchmarks for the sake of algorithm testing.

Reporting for specific materials, systems and methods

We require information from authors about some types of materials, experimental systems and methods used in many studies. Here, indicate whether each material, system or method listed is relevant to your study. If you are not sure if a list item applies to your research, read the appropriate section before selecting a response.

Materials & experimental systems

n/a	Involved in the study
☒	☐ Antibodies
☒	☐ Eukaryotic cell lines
☒	☐ Palaeontology and archaeology
☒	☐ Animals and other organisms
☒	☐ Clinical data
☒	☐ Dual use research of concern
☒	☐ Plants

Methods

n/a	Involved in the study
☒	☐ ChIP-seq
☒	☐ Flow cytometry
☒	☐ MRI-based neuroimaging

Plants

Seed stocks	*Report on the source of all seed stocks or other plant material used. If applicable, state the seed stock centre and catalogue number. If plant specimens were collected from the field, describe the collection location, date and sampling procedures.*
Novel plant genotypes	*Describe the methods by which all novel plant genotypes were produced. This includes those generated by transgenic approaches, gene editing, chemical/radiation-based mutagenesis and hybridization. For transgenic lines, describe the transformation method, the number of independent lines analyzed and the generation upon which experiments were performed. For gene-edited lines, describe the editor used, the endogenous sequence targeted for editing, the targeting guide RNA sequence (if applicable) and how the editor was applied.*
Authentication	*Describe any authentication procedures for each seed stock used or novel genotype generated. Describe any experiments used to assess the effect of a mutation and, where applicable, how potential secondary effects (e.g. second site T-DNA insertions, mosiacism, off-target gene editing) were examined.*
